# Inhibition of mitochondrial complex I induces mitochondrial ferroptosis by regulating CoQH2 levels in cancer

**DOI:** 10.1038/s41419-025-07510-6

**Published:** 2025-04-05

**Authors:** Ru Deng, Lingyi Fu, Haoyu liang, Xixiong Ai, Fangyi Liu, Nai Li, Liyan Wu, Shuo Li, Xia Yang, Yansong Lin, Yuhua Huang, Jingping Yun

**Affiliations:** 1 State Key Laboratory of Oncology in South China, Collaborative Innovation Center for Cancer Medicine, Sun Yat-sen University Cancer Center, Guangzhou, China; 2 Department of Pathology, Sun Yat-sen University Cancer Center, Guangzhou, China; 3 State Key Laboratory of Oncology in South China, Guangdong Provincial Clinical Research Center for Cancer, Sun Yat-sen University Cancer Center, Guangzhou, China; 4 Department of Radiation Oncology, Peking University Shenzhen Hospital, Shenzhen, China; 5 Reproductive Medicine Center, The Affiliated Shenzhen Maternity and Child Healthcare Hospital of the South Medical University, Shenzhen, Guangdong China

**Keywords:** Cell death, Cancer metabolism, Targeted therapies

## Abstract

Ferroptosis, a novel form of regulated cell death induced by the excessive accumulation of lipid peroxidation products, plays a pivotal role in the suppression of tumorigenesis. Two prominent mitochondrial ferroptosis defense systems are glutathione peroxidase 4 (GPX4) and dihydroorotate dehydrogenase (DHODH), both of which are localized within the mitochondria. However, the existence of supplementary cellular defense mechanisms against mitochondrial ferroptosis remains unclear. Our findings unequivocally demonstrate that inactivation of mitochondrial respiratory chain complex I (MCI) induces lipid peroxidation and consequently invokes ferroptosis across GPX4 low-expression cancer cells. However, in GPX4 high expression cancer cells, the MCI inhibitor did not induce ferroptosis, but increased cell sensitivity to ferroptosis induced by the GPX4 inhibitor. Overexpression of the MCI alternative protein yeast NADH-ubiquinone reductase (NDI1) not only quells ferroptosis induced by MCI inhibitors but also confers cellular protection against ferroptosis inducers. Mechanically, MCI inhibitors actuate an elevation in the NADH level while concomitantly diminishing the CoQH2 level. The manifestation of MCI inhibitor-induced ferroptosis can be reversed by supplementation with mitochondrial-specific analogues of CoQH2. Notably, MCI operates in parallel with mitochondrial-localized GPX4 and DHODH to inhibit mitochondrial ferroptosis, but independently of cytosolically localized GPX4 or ferroptosis suppressor protein 1(FSP1). The MCI inhibitor IACS-010759, is endowed with the ability to induce ferroptosis while concurrently impeding tumor proliferation in vivo. Our results identified a ferroptosis defense mechanism mediated by MCI within the mitochondria and suggested a therapeutic strategy for targeting ferroptosis in cancer treatment.

## Introduction

Ferroptosis, distinct from apoptosis, necrosis, or autophagy, represents an emerging paradigm of regulated cell death characterized by the excessive accumulation of lipid peroxidation products in an iron-dependent manner [1–3]. The accruing body of evidence underscores the substantial linkage between ferroptosis and human diseases, assuming a pivotal function in inhibiting tumor progression [4, 5]. p53 induces ferroptosis by downregulating the transcription of SLC7A11, thereby inhibiting tumor proliferation both in vivo and in vitro [3]. Concomitantly, the tumor suppressor BRCA1-associated Protein 1 (BAP1), exerts a promotive influence on ferroptosis while concurrently impeding tumor advancement through the attenuation of SLC7A11 expression [6]. Under harsh environmental stress conditions, malignant cells are predisposed to undergo ferroptosis due to the perturbed equilibrium of reactive oxygen species (ROS), iron ions, and polyunsaturated fatty acids [7]. However, tumor cells incorporate three pivotal systems that collectively counteract ferroptosis. The XC-GPX4-GSH system: cystine/glutamate reverse transporter (system XC) mediates the intracellular influx of cystine, which synthesizes GSH in the cytoplasm; GPX4 enzyme utilizes GSH as a pivotal cofactor to catalyze the reduction of lipid peroxides into alcohols, thus protecting cells against ferroptosis. In mammalian cells, several isoforms of GPX4, including cytosolic and mitochondrial isoforms, are encoded by a single gene. The mitochondrial isoform of GPX4 is believed to localize in mitochondria owing to the presence of a mitochondrial targeting signal (MTS). However, this MTS-GPX4 isoform is physiologically expressed in the testis and optic nerve, with minimal expression in other tissues or cultured cells. The cytosolic GPX4 isoform can cross the mitochondrial outer membrane and accumulate in the intermembrane space, where it helps to suppress mitochondrial lipid peroxidation [8–11]. In our study, mitoGPX4 represent an artificial experimental tool model, aimed at exploring the function of these isoforms in mitochondrial ferroptosis rather than mimicking physiological events. The FSP1-CoQH2 system, ferroptosis suppressor protein 1 (FSP1), operates as an NAD(P)H-dependent oxidoreductase and mediates the reduction of CoQ to CoQH2, which captures and decomposes lipid peroxidation products, thereby contributing to ferroptosis defence [12, 13]. The DHODH-CoQH2 system is dihydroorotate dehydrogenase (DHODH), an enzyme pivotal in pyrimidine biosynthesis, which assumes the role of reducing CoQ to CoQH2 within the mitochondrial inner membrane. This enzymatic modulation serves to dismantle lipid peroxides within the mitochondria and effectively suppresses ferroptosis [14, 15].

As the main regulator of oxidative phosphorylation (OXPHOS), mitochondria are the main intracellular ROS producers [16]. Mitochondria are also the centers of iron metabolism [17]. A growing number of studies have reported the roles of mitochondria and ETC complexes in ferroptosis in various cancer models with different conclusions [18–21]. This may be due to the highly context-dependent nature of ferroptosis [22, 23]. In conclusion, mitochondria indisputably play a pivotal role in oxidative metabolism; however, their involvement in ferroptosis remains unclear.

Mitochondrial respiratory chain complexes I, II, III, IV, and V (MCI-MCV) collectively orchestrate electron transportation for adenosine triphosphate (ATP) synthesis, a cardinal process vital to cellular function [24]. In mitochondria, MCI oxidizes NADH from the tricarboxylic acid cycle or cytosol, reduces CoQ which from mitochondria, and transports protons across the inner membrane, contributing to the proton-motive force [25]. While normal cells predominantly rely on mitochondrial oxidative phosphorylation for energy supply, malignant cells rely on aerobic glycolysis for sustenance, a phenomenon termed the Warburg effect [26, 27]. However, accumulating evidence underscores the indispensable role of mitochondria, specifically MCI, in tumor survival [28–30].

In this study, we found that MCI operates in parallel with mitoGPX4 and DHODH(but independently of cytoGPX4 or FSP1) to inhibit ferroptosis in the mitochondrial inner membrane by reducing CoQ to CoQH2 in GPX4 low expression cancer cells. In addition, MCI inhibitors manifest the potential to elicit tumor-specific ferroptosis in vivo. The present study unveils the prospective contribution and molecular mechanisms through which MCI protects cells against ferroptosis in the inner mitochondrial membrane. These findings offer valuable insights into the elucidation of novel pharmacotherapeutic strategies for new mechanisms of tumor-associated ferroptosis.

## Materials and methods

### Cell lines and reagents

The SKOV3, PLC/PRF/5, ES-2, HT-1080, HCT-116, NCI-H23, and NCI-H1650 cell lines were obtained from the American Type Culture Collection (ATCC). Huh7 cells were obtained from the Cell Bank of the Chinese Academy of Sciences (Shanghai, China). Each cell line was then subjected to STR-based identification and was not contaminated by mycoplasma. Huh7, PLC/PRF/5, ES-2, HT-1080, and HCT-116 cells were cultured in Dulbecco’s modified Eagle’s medium (DMEM) supplemented with 10% heat-inactivated fetal bovine serum (FBS), penicillin (100 μg/mL), and streptomycin (100 μg/mL). These cultures were maintained in a humidified incubator with an atmosphere of 5% CO_2_ at a temperature of 37 °C. On the other hand, NCI-H23 and NCI-H1650 cells were cultured using RPMI 1640 medium.

RSL3(MedChemExpress), ML162(MedChemExpress), rotenone (MedChemExpress), IACS-010759(MedChemExpress), Brequinar (MedChemExpress), Ferrostatin-1 (MedChemExpress), Z-VAD-FMK (MedChemExpress), Tempo (MedChemExpress), MitoTempo (Sigma-Aldrich), MitoQ (Cayman Chemical), MitoQH2 (Cayman Chemical),4-CBA(Sigma-Aldrich), BODIPY 581/591C11(Invitrogen), and mito-BoDIPY (GLPBIO).

### Plasmid construction

In the context of mitoGPX4, cytoGPX4, mitoFSP1, cytoFSP1, or AOX-Flag overexpression, the pertinent coding sequences (CDS) were meticulously amplified and subsequently inserted into the plvx-puro lentivirus vector. mitoGPX4 is constructed by adding a N-terminus mitochondrial leading sequence to cytosolic GPX4 isoform. N-terminus mitochondrial leading sequence for constructing mito-GPX4/FSP1: ATGAGCCTCGGCCGCCTTTGCCGCCTACTGA AGCCGGCGCTGCTCTGTGGGGCTCTG GCCGCGCCTGGCCTGGCCGGGACC. For expressing GPX4, the selenocysteine insertion sequence included in the 3’UTR: CTCCACAAGTGTGTGGCCCCGCCCGAGCCCCTGCCCA CGCCCTTGGAGCCTTCCACCGGCACTCATGACGGCCTGCCTGCAAACCTGCTGGTGGGGCAGACCCGAAAATCCAGCGTGCACCCCGCCGGAGGAAGGTCCCATGGCCTGCTGGGCTTGGCTCGGCGCCCCCACCCCTGGCTACCTTGTGGGAATAAACAGACAAATTAGCCTGCTGGATAAAAAAA. For the explicit purpose of NDI1-Flag overexpression, the CDS were subjected to thorough amplification and subsequent integration into the psin-Flag-puro lentivirus vector, with FLAG tag inserted at the N-terminus. In pursuit of DHODH overexpression, CDS underwent comprehensive amplification prior to their successful incorporation into the LV105-puro lentivirus vector. In instances involving knockdown of DHODH, short hairpin RNAs (shRNAs) were introduced and integrated into the pLKO.1 puro lentiviral vector. In the case of FSP1 knockout, indispensable sgRNA oligos were meticulously inserted into the lentiCRISPR v2 lentiviral vector. It is pertinent to note that the specific target sequences for both shRNA and sgRNAs have been exhaustively documented and are available in Supplementary Table 1 for reference.

### Cell viability assay

Cells were seeded into 96-well flat-bottomed plates and subjected to drug treatments of the appropriate duration on the subsequent day. Following the treatment period, cell viability was assessed using the Cell Counting Kit-8 (CCK-8, APExBIO). Specifically, 10 μL of CCK-8 reagent was added to each well, and the cells were incubated at 37 °C for 1 h. Subsequently, the absorbance at 450 nm was measured using a microplate reader (Tecan Infinite 200 Pro). Huh7 cells were treated with ROT (50 μM) for 10 h, whereas NCI-H23 cells were exposed to ROT (50 μM) for 14 h. As for cells subjected to IACS-010759 treatment, this intervention was carried out for 48 h.

### Lipid peroxidation measurement

Cells were seeded into 12-well plates and subjected to drug treatment on the following day for a duration deemed appropriate. Subsequently, cells were exposed to 5 μM C11-BODIPY 581/591 (Invitrogen). Following the incubation, a 30 min period was allotted at 37 °C. The cells were thoroughly washed with 1 mL of PBS and subsequently subjected to trypsin digestion to yield a cellular precipitate. The precipitate was subsequently resuspended in 300 μL of PBS. Changes in fluorescence were systematically analyzed using a CytoFLEX flow cytometer (Beckman). All experiments were conducted in triplicate to ensure robustness and consistency. In specific instances, such as with Huh7, ES-2, and HCT-116 cells, ROT (50 μM) treatment was administered for 8 h. Similarly, NCI-H23, PLC/PRF/5, NCI-H1650, and HT-1080 cells were exposed to ROT (50 μM) for an extended interval of 12 h. Additionally, cells were subjected to IACS-010759 treatment at a concentration of 50 μM for 48 h.

### NAD+ and NADH measurement

The samples and NADH standards were prepared in strict adherence to the manufacturer’s guidelines, as provided by Beyotime. Subsequently, 20 μL of the prepared sample was added to 96-well flat-bottom plates. The reaction was subjected to an incubation at 37 °C, followed by the quantification of absorbance at a wavelength of 450 nm. The cells were treated with RSL3 (10 μM), ML162 (10 μM), NADH (1 mM), ROT (50 μM), BQR (50 μM), mitoQH2(5 μM), and MitoTempo(5 μM).

### Mitochondrial morphology measurement

To conduct measurements pertaining to mitochondrial morphology, cells underwent a thorough washing procedure using PBS for three times. Following this, the cells were subjected to treatment with 500 nM PK Mito Deep Red (PKMDR-2, Genvivo) at 37 °C for 30 min. This was followed by three additional washes with FluoroBrite™ DMEM. Images were obtained and analyzed using a SIMSTORM microscope (N-SIM/N-STORM, Nikon).

### Transmission electron microscopy

Huh7 cells were treated with ROT (50 μM) for 8 h or with IACS-010759 (50 μM) for 48 h. Similarly, NCI-H23 cells were subjected to ROT treatment (50 μM) for 12 h or alternatively, treated with IACS-010759 (50 μM) for 48 h. Following treatment, the cells were collected and fixed. In subsequent steps, the cells were dehydrated using an ethanol gradient. The treated cells were carefully embedded in resin medium. Subsequently, the resin blocks containing the embedded cells were precision-cut into thin sections, measuring 60-80 nm in thickness. These sections were meticulously retrieved and placed on cuprum grids with a mesh size of 150. Thin sections were stained with uranium acetate saturated alcohol. Subsequent examinations were performed using transmission electron microscopy (HITACHI).

### CoQ and CoQH2 analysis

Cells were cultivated in 10 cm plates until they reached a confluence of approximately 70%. After 8-h of treatment with ROT (50 μM), the cells were subjected to a single wash using 5 mL PBS. The cells were subjected to trypsin digestion, followed by cell counting. The cells were then centrifuged at 4 °C and the supernatant was discarded. A spike was introduced into the cell samples, consisting of 200 μL of 96% ethanol supplemented with BHT and 800 μL of 1-propanol. The cell samples were then subjected to five cycles of “sonication for 1 min and rest for 1 min”. Subsequently, the samples were allowed to rest at −40 °C for 30 min, followed by an additional rest at 4 °C for 10 min. The samples were then centrifuged at 4 °C (15 min, 15000 rcf) and the resulting precipitate was dried using mild nitrogen. The dried residue was subsequently reconstituted in 50 μL ethanol containing the internal standard for UHPLC-MS/MS analysis. UHPLC-MS/MS analysis was conducted using an Agilent 1290 Infinity II UHPLC system coupled with a 6470 A Triple Quadrupole mass spectrometer (Santa Clara, CA, United States). Samples were injected onto a Thermo Fisher Acclaim C30 column (100 mm × 2.1 mm, 3 μm) at a flow rate of 0.3 mL/min. The mobile phases consisted of (A) 60% acetonitrile and (B) isopropanol/acetonitrile (9:1, v/v), both of which contained 10 mM ammonium formate. Chromatographic separation was achieved through a gradient elution program as follows: 10% B from 0 to 0.5 min, 70% B at 1.5 min, 90% B at 8 min, 100% B at 8.5 min and held until 9.5 min, and 10% B at 9.6 min, held until 11 min. The raw data were processed using MassHunter Workstation Software (version B.08.00, Agilent) with default parameters, with manual inspection for quality assurance of both the qualitative and quantitative aspects of each compound. The integrated peak areas of each compound in all the samples were determined. Calibration curves were established using an 11-point calibration approach, which involved plotting the peak area ratios of each compound to the internal standard against the concentration of the respective compound. Initially, the molar amount of CoQ (oxidized CoQ10) in the CoQH2 (reduced CoQ10) sample was calculated using the calibration curve of CoQ. The true molar concentration of CoQH2 was determined by subtracting the molar concentration of CoQ from the nominal value of CoQH2. This true concentration was then used to construct calibration curves for CoQH2.

### RNA isolation and real-time quantitative PCR (RT-qPCR)

Total RNA was extracted using the TRIzol reagent (Invitrogen). After extraction, reverse transcription was performed using the HiScript II Q RT SuperMix Kit (Vazyme Biotech, Nanjing, China). qRT–PCR was performed using SYBR GreenER qPCR SuperMix Universal (Invitrogen) on a CFX96 real-time PCR detection system (Bio-Rad). The primer sequences used for these analyses are listed in Supplementary Table 1. The cells were treated with ROT(50 μM), IACS(50 μM), RSL3 (1 μM), ML162 (1 μM), Fer-1 (10 μM), ZVF (10 μM), BQR (50 μM), QH2(5 μM), mitoQH2(5 μM), tempo (5 μM), and MitoTempo(5 μM).

### Western blotting

Western blotting was performed as previously described [31]. The following antibodies were used: GPX4 (67763-1, Proteintech), FSP1 (sc-377120, SANTA), DHODH (14877-1, Proteintech), SLC7A11 (98051, CST), vinculin (66305-1, Proteintech), a-Tubulin(11224-1, Proteintech), VDAC (4661, CST), NDUFS4 (sc-100567, SANTA) and Flag (14793, CST).

### Histology and immunohistochemistry

The method was performed as described previously [31]. Briefly, freshly collected tumor tissues were fixed overnight in formalin. These tissues were subsequently sectioned into slides measuring 4-5 mm in thickness and designated for hematoxylin and eosin (H&E) staining or immunohistochemistry (IHC) analysis. Antibodies used in this analysis were GPX4 (67763-1, Proteintech), cleaved caspase-3 (9661, CST), ki67 (ab15580, Abcam) and 4-HNE (ab46545, Abcam). Immunohistochemistry (IHC) analysis was performed as previously described [31].

### Cell line-derived xenograft models

All animal procedures were approved by the Institutional Animal Care and Use Committee of the Sun Yat-sen University (L102012023004H). Female BALB/c nude mice, aged 4–6 weeks, were procured from the Guangdong Medical Lab Animal Center. The animal sex had no effect on the conclusions of this study. The investigator was blinded to the group allocation of the animals during the experiment. No statistical method was used to predetermine the sample size for the xenograft mice experiment, which was based on previous experimental observations. All animals used in this study were chosen randomly. Cancer cell lines were suspended and quantified in cold PBS, and 5 × 10^6^ Huh7 or NCI-H23 cells were subcutaneously inoculated into the mice. After 2 weeks, the mice received oral dosing IACS-010759 (5 mg/kg/day) following a 5 on/2 off dosing schedule until the study endpoint. After treatment initiation, tumor volume measurements were recorded every two days until the study endpoint, with calculations performed using the following formula: volume = length × width^2^ × 1/2. The body weights of the mice were measured every three days. Upon completion of each experiment, tumors were carefully removed, photographed, and weighed. All experimental procedures were conducted in strict accordance with the guidelines approved by the Ethics Committee of the Animal Experimental Center of Sun Yat-sen University. The ethical node of the animal experiments in this study was L102042025020F.

### Statistics and reproducibility

All sample sizes were determined to ensure robust statistical analyses, which were conducted using GraphPad Prism 8.0 (GraphPad Software). All statistical analyses were performed using an unpaired two-sided *t*-test analysis of variance. For statistical significance indicators, the conventions of *P < 0.05, **P < 0.01, ***P < 0.001, and ****P < 0.0001 were employed. Instances in which statistical significance was not observed were denoted as “ns” for non-significant. The outcomes of the cell culture experiments were obtained from a minimum of three independent replicates for each experimental condition.

## Results

### MCI regulates ferroptosis by utilizing NADH independently of FSP1 in tumors

**Fig. 1:**
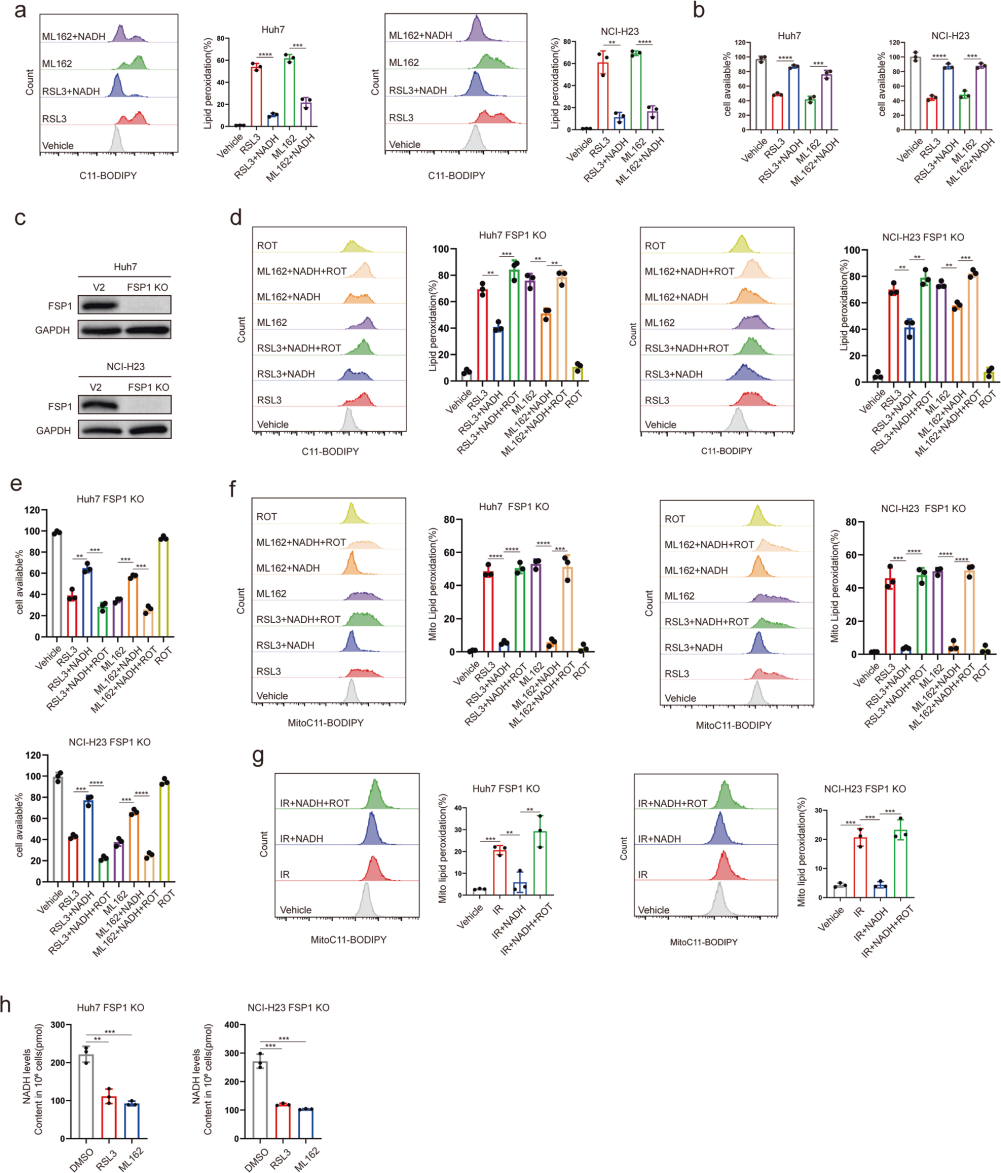
MCI regulates ferroptosis by utilizing NADH independently of FSP1 in tumors. **a** Lipid peroxidation levels in Huh7 and NCI-H23 cells treated with RSL3(1 μM) or ML162(1 μM) for 1.5 h after pretreatment with NADH(1 mM) for 24 h. **b** Cell viability of Huh7 or NCI-H23 cells treated with RSL3 (1 μM) or ML162 (1 μM) for 2 h after pretreatment with NADH(1 mM) for 24 h. **c** FSP1 protein levels in FSP1 KO Huh7 or NCI-H23 cells. **d** Lipid peroxidation levels in Huh7 or NCI-H23 FSP1 KO cells treated with RSL3 (0.5 μM) or ML162 (0.5 μM) for 1.5 h, with or without Rotenone (ROT; 50 μM) for 2 h, following pretreatment with NADH (1 mM) for 24 h. **e** Cell viability of Huh7 or NCI-H23 cells treated with RSL3 (0.5 μM) or ML162 (0.5 μM), with or without ROT (10 μM) for 8 h, following pretreatment with NADH (1 mM) for 24 h. **f** Mitochondrial lipid peroxidation levels in Huh7 or NCI-H23 FSP1 KO cells treated with RSL3 (0.5 μM) or ML162 (0.5 μM) for 1.5 h, with or without Rotenone (ROT; 50 μM) for 2 h, following pretreatment with NADH (1 mM) for 24 h. **g** Mitochondrial lipid peroxidation in FSP1-KO cells 24 h after exposure to 10 Gy of IR, with or without ROT (50 μM) for 2 h, following pretreatment with NADH (1 mM) for 24 h. **h** NADH levels in Huh7 or NCI-H23 FSP1 KO cells treated with RSL3 (10 μM) or ML162 (10 μM) for 2 h.

NAD(P)H serves as a substrate for FSP1 in the reduction of CoQ to CoQH2, CoQH2 captures and decomposes lipid peroxidation products, thereby contributing to the ferroptosis defence [13]. NADH has demonstrated the capability attenuated lipid peroxidation and ferroptosis induced by class II ferroptosis inducers RSL3 or ML162 in both Huh7 and NCI-H23 cells (Fig. 1a, b). Subsequently, we conducted a knockout (KO) of FSP1 in Huh7 and NCI-H23 cells (Fig. 1c), which revealed that NADH retained its ability to attenuate RSL3- or ML162-induced lipid peroxidation and ferroptosis (Fig. 1d, e). In mammalian tissues, cellular redox and bioenergetic states are regulated by metabolic NADH shuttles that facilitate the transfer of NADH across the inner mitochondrial membrane into the mitochondria for oxidation [32]. The major shuttles are the a-glycerophosphate shuttle and the malate–aspartate shuttle (MAS). NADH is membrane-impermeable, but MAS could transport cytosolic NADH into the mitochondrial matrix, maintaining higher NADH/NAD ratio in the mitochondria than in the cytosol [33, 34]. We detected increased NADH levels in the mitochondria of FSP1-KO cells treated with NADH (Supplementary Fig. S1a). These results suggest that NADH inhibits ferroptosis via pathways that are independent of FSP1. NADH participates in various biochemical reactions, including the reduction of CoQ to CoQH2 [25, 35], conversion of pyruvate into lactate [36], transport of oxaloacetic acid out of mitochondria [37], gluconeogenesis [38], and electron transport within the MCI [25] process, wherein the MCI process consumes the most NADH. Consequently, we posit that the resistance to ferroptosis, mediated by NADH in FSP1-KO Huh7 and NCI-H23 cells, is likely connected to the MCI process occurring in mitochondria. Mito-BoDIPY staining (a mitochondria-targeted variant of C11-BoDIPY that selectively detects mitochondrial lipid peroxidation) demonstrated that NADH mitigate RSL3- or ML162-induced mitochondrial lipid peroxidation (Fig. 1f). Radiotherapy can induce lipid peroxidation by generating large amounts of ROS, which ultimately leads to ferroptosis [39]. Mito-BoDIPY staining revealed that NADH effectively attenuated the amplified mitochondrial lipid peroxidation induced by radiotherapy in FSP1-KO cells (Fig. 1g). Upon treatment with RSL3 or ML162, an obvious reduction in NADH levels was evident in FSP1-KO Huh7 and NCI-H23 cells (Fig. 1h), suggesting MCI’s role in diminishing lipid peroxidation via the utilization of NADH in the reduction of CoQ to CoQH2. Rotenone, recognized as an MCI inhibitor, interferes with the electron transport of NADH [40]. The application of rotenone effectively reversed the NADH-mediated suppression of lipid peroxidation or mitochondrial lipid peroxidation and ferroptosis triggered by RSL3 or ML162 in FSP1-KO Huh7 and NCI-H23 cells (Fig. 1d–f). Correspondingly, NADH-mediated suppression of mitochondrial lipid peroxidation induced by radiotherapy in FSP1-KO cells was counteracted by rotenone treatment (Fig. 1g). Collectively, these results emphasize that MCI exerts an independent influence on ferroptosis in tumors through its reliance on NADH independent of FSP1.

### Inhibition of MCI induces ferroptosis

**Fig. 2:**
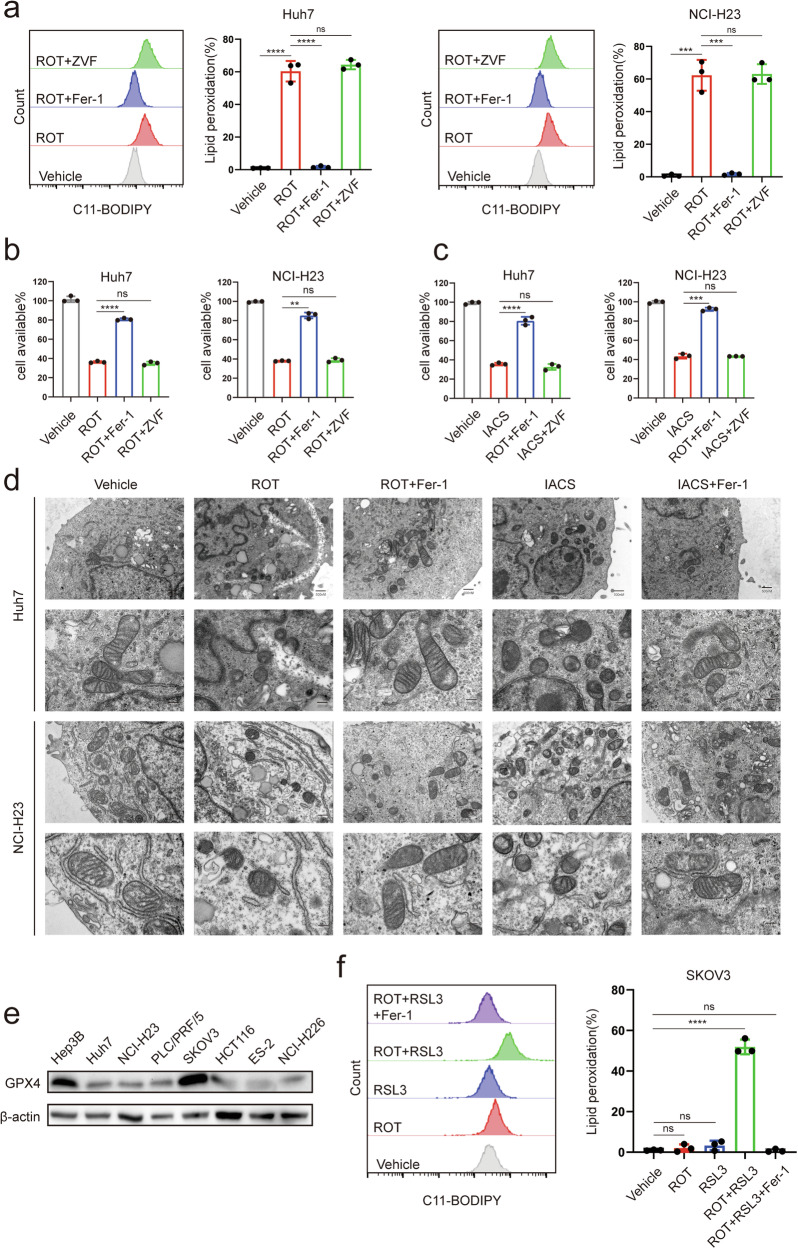
Inhibition of MCI induces ferroptosis. **a** Lipid peroxidation levels in Huh7 or NCI-H23 cells treated with ROT (50 μM), following pretreatment with Ferrostatin-1 (Fer-1; 10 μM) or Z-VAD-FMK (ZVF; 10 μM). **b** Cell viability in Huh7 or NCI-H23 cells treated with ROT (50 μM) after pretreatment with Fer-1 (10 μM) or ZVF(10 μM). **c** Cell viability in Huh7 or NCI-H23 cells treated with IACS-010759(IACS ;50 μM) after pretreatment with Fer-1 (10 μM) or ZVF (10 μM). **d** Mitochondrial morphology observed by transmission electron microscopy in Huh7 cells treated with ROT (50 μM) or IACS (50 μM) after pretreatment with Fer-1 (10 μM). **e** Protein levels of GPX4 in different cancer cell. **f** Lipid peroxidation levels in SKOV3 cells treated with ROT (50 μM) or RSL3(0.5 μM), following pretreatment with Fer-1(10 μM).

We hypothesized that MCI is associated with mitochondrial ferroptosis, whether inhibiting MCI directly affects ferroptosis? Upon subjecting Huh7 and NCI-H23 cells to treatment with the MCI inhibitor rotenone or IACS-010759, an obvious elevation in lipid peroxidation and augmented expression of the ferroptosis-associated marker gene PTGS2 was observed, concomitant with a reduction in cell viability (Fig. 2a–c and Supplementary Fig. S1b–f). Multiple studies have shown that MCI inhibitors can attenuate aspartate synthesis, resulting in the restriction of tumor proliferation, but pyruvate can stimulate aspartate synthesis [30, 41–43]. In the subsequent phase of our investigation, rotenone or IACS-010759 treatment was applied with pyruvate. However, the supplementary administration of aspartate or pyruvate did not counteract the heightened lipid peroxidation induced by rotenone or IACS-010759 (Supplementary Fig. S1g). Notably, the increased lipid peroxidation and PTGS2 expression, as well as the decreased cell viability induced by rotenone or IACS-010759, were effectively mitigated by the application of the ferroptosis inhibitor Ferrostatin-1. Conversely, treatment with the apoptosis inhibitor ZVAD-FMK was ineffective (Fig. 2a–c and Supplementary Fig. S1d–f). Rotenone or IACS-010759 administration contributed to ferroptosis -like morphological changes in mitochondria of Huh7 and NCI-H23 cells (Fig. 2d and Supplementary Fig. S1h). In general, cells remain resistant to ferroptosis, as long as they maintain sufficient levels of GPX4. Whether MCI inhibitor-mediated ferroptosis in Huh7 and NCI-H23 cells is related to GPX4 expression? We detected GPX4 expression in several tumor cells and found that GPX4 expression was low in Huh7 and H23 cells, whereas the opposite was observed in SKOV3 and Hep3B cells (Fig. 2e). Intriguingly, rotenone did not induce ferroptosis in SKOV3 or Hep3B cells, but increased its ferroptosis sensitivity to the GPX4 inhibitor (Fig. 2f and Supplementary Fig. S1i). Notably, the administration of rotenone resulted in augmented lipid peroxidation in various GPX4 low-expression cancer cell lines, which could be reversed by the application of ferroptosis inhibitors (Supplementary Fig. S2a). Remarkably, treatment with rotenone and IACS-010759 did not elicit discernible changes in the mRNA or protein levels of GPX4, FSP1, DHODH, or SLC7A11 in Huh7 and NCI-H23 cells (Supplementary Fig. S2b, c). NDUFS4 is an important subunit of MCI, and its abnormality can lead to dysfunction of MCI [44]. Knockdown of NDUFS4(Supplementary Fig. S2d) induces ferroptosis which can be alleviated by Ferrostatin-1(Supplementary Fig. S2e, f). Collectively, these findings indicate that inhibition of MCI serves as a trigger for ferroptosis in GPX4 low-expression tumor cells.

### MCI protects cells against ferroptosis in mitochondria

**Fig. 3:**
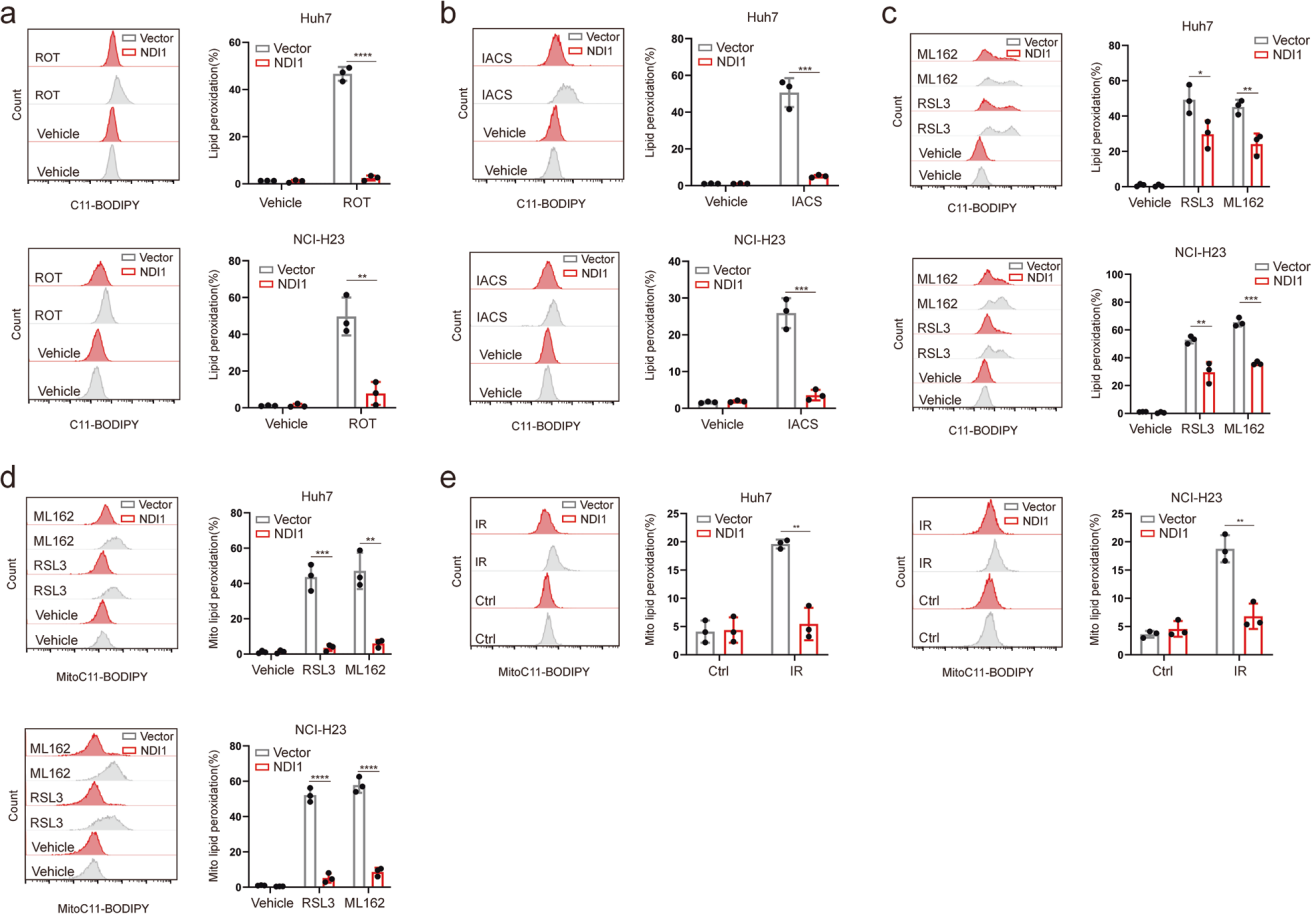
MCI protects cells against ferroptosis in mitochondria. Lipid peroxidation levels in NDI1 overexpression Huh7 or NCI-H23 cells treated with ROT (50 μM) (**a**) or IACS (50 μM) (**b**). **c** Lipid peroxidation levels in NDI1 overexpression Huh7 or NCI-H23 cells treated with RSL3 (0.5 μM) or ML162 (0.5 μM) for 1.5 h. **d** Mitochondrial lipid peroxidation in NDI1 overexpression Huh7 or NCI-H23 cells treated with RSL3 (0.5 μM) or ML162 (0.5 μM) for 1.5 h. **e** Mitochondrial lipid peroxidation in NDI1 overexpression Huh7 or NCI-H23 cells 24 h after exposure to 10 Gy of IR.

Saccharomyces cerevisiae alternative NADH dehydrogenase (NDI1), which bound to the mitochondrial inner membrane facing the matrix site mediates the coupling of NADH oxidation to NAD+ to reduce CoQ to CoQH2 without generating a proton-motive force, thus restore the electron transport activity of mitochondrial complex I [45–47].we ectopically expressed NDI1 in both Huh7 and NCI-H23 cells (Supplementary Fig. S3a). The overexpression of NDI1 in Huh7 and NCI-H23 cells suppressed ferroptosis induced by rotenone or IACS-010759 (Fig. 3a, b and Supplementary Fig. S3b, c), suggesting that ferroptosis induced by rotenone or IACS-010759 in Huh7 and NCI-H23 cells is not due to the non-specific effects of these inhibitors but involves electron transport of MCI. Furthermore, ferroptosis induced by RSL3 or ML162 was partially restored by the overexpression of NDI1 in Huh7 and NCI-H23 cells (Fig. 3c and Supplementary Fig. S3d). Mito-BoDIPY staining illustrated that Huh7 and NCI-H23 cells overexpressing NDI1 exhibited more pronounced resistance against mitochondrial lipid peroxidation induced by RSL3 or ML162 (Fig. 3d), indicating that MCI mainly resists mitochondrial ferroptosis. Overexpression of NDI1 in Huh7 and NCI-H23 cells suppressed mitochondrial lipid peroxidation induced by rotenone or IACS-010759 treatment (Supplementary Fig. S3e, f). Additionally, Mito-BoDIPY staining demonstrated that overexpression of NDI1 in Huh7 and NCI-H23 cells effectively prevented mitochondrial ferroptosis induced by radiotherapy (Fig. 3e). In summary, our findings collectively highlight the protective potential of MCI against mitochondrial ferroptosis.

### MCI inhibits ferroptosis by regulating CoQH2 levels

**Fig. 4:**
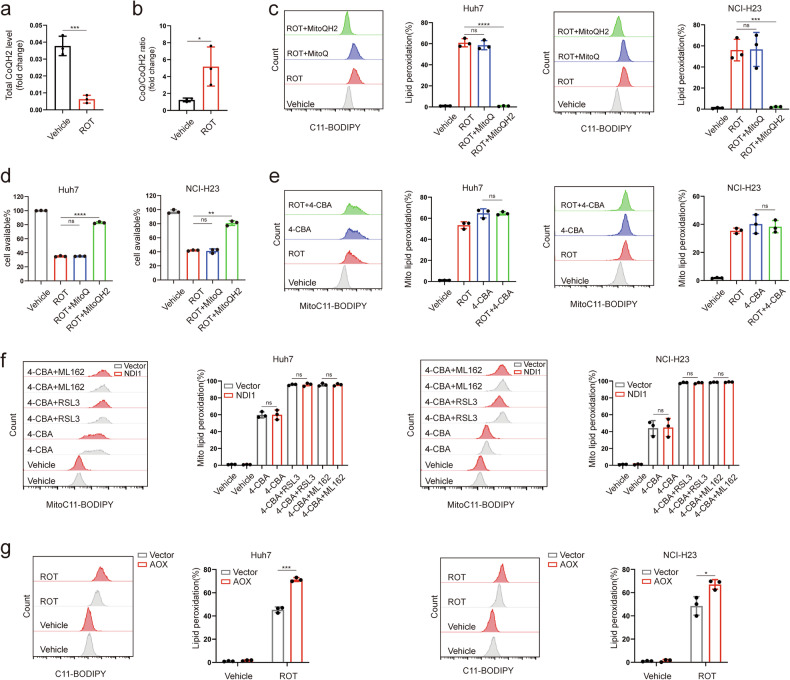
MCI inhibits ferroptosis by regulating CoQH2 levels. **a** Total CoQH2 levels in Huh7 cells treated with R ROT (50 μM) for 8 h. **b** Ubiquinone/CoQH2 ratio in Huh7 cells treated with ROT (50 μM) for 8 h. **c** Lipid peroxidation levels in Huh7 and NCI-H23 cells treated with ROT(50 μM), following pretreatment with MitoQ (5 μM) or MitoQH2 (5 μM). **d** Cell viability in Huh7 or NCI-H23 cells treated with ROT, following pretreatment with MitoQ (5 μM) or MitoQH2 (5 μM). **e** Lipid peroxidation levels in Huh7 and NCI-H23 cells treated with ROT (50 μM) with or without 4-CBA(5 mM) for 24 h. **f** Lipid peroxidation levels in NDI1 overexpression Huh7 or NCI-H23 cells treated with RSL3 (0.5 μM) or ML162 (0.5 μM) for 1.5 h, following treatment with 4-CBA (5 μM) for 24 h. **g** Lipid peroxidation levels in AOX-overexpressing Huh7 or NCI-H23 cells treated with ROT(50 μM).

MCI facilitated the reduction of CoQ to CoQH2 by utilizing electrons derived from NADH [46]. In light of this mechanism, our hypothesis centered on MCI inhibitors that provoke ferroptosis within tumor cells through a reduction in CoQH2 levels. Upon administration of rotenone, a discernible reduction in the CoQH2 content accompanied by an increase in the CoQ/CoQH2 ratio was observed (Fig. 4a, b). Notably, the application of mitochondria-targeted analogs of CoQ and CoQH2, Mitoquinone (MitoQ) and Mitoquinol (MitoQH2), respectively, revealed that MitoQH2 alone was effective in defending rotenone-induced ferroptosis in Huh7 and NCI-H23 cells, with no discernible impact observed in MitoQ treatment (Fig. 4c, d and Supplementary Fig. S4a). The radical-trapping antioxidant 2,2,6,6-tetramethylpiperidinyl-1-oxy (Tempo) can trap radicals and suppress ferroptosis [14]. Treatment with mitochondrion-targeted TEMPO (mitoTempo) suppressed rotenone-mediated ferroptosis, whereas tempo had no effect (Supplementary Fig. S4b-d).

The obstruction of CoQ biosynthesis by 4-chlorobenzoic acid (4-CBA) led to a notable reduction in total CoQ levels [14]. Treatment with 4-CBA induces ferroptosis in Huh7 and NCI-H23 cells. Interestingly, rotenone alone did not further exacerbate ferroptosis or NADH level following 4-CBA treatment (Fig. 4e and Supplementary Fig. S4e-g), indicating that the MCI inhibitor induced ferroptosis by regulating the CoQH2/CoQ levels. 4-CBA effectively reversed the resistance to mitochondrial lipid peroxidation engendered by NDI1 overexpression in Huh7 and NCI-H23 cells (Fig. 4f), suggesting that NDI1 protects against mitochondrial ferroptosis by regulating CoQH2/CoQ levels. Introducing Ciona intestinalis AOX (AOX), an enzyme capable of directly transporting electrons from CoQH2 to oxygen, thereby reducing CoQH2 levels and bypassing the activities of ETC complexes III and IV [46]. AOX overexpression had a distinct sensitizing effect on rotenone-induced ferroptosis in Huh7 and NCI-H23 cells (Fig. 4g and Supplementary Fig. S4h). These observations collectively underscore the pivotal role of MCI in suppressing ferroptosis within the mitochondria through the reduction of CoQ to CoQH2.

### MCI acts in parallel with mitochondrial GPX4 and DHODH to suppress mitochondrial ferroptosis

**Fig. 5:**
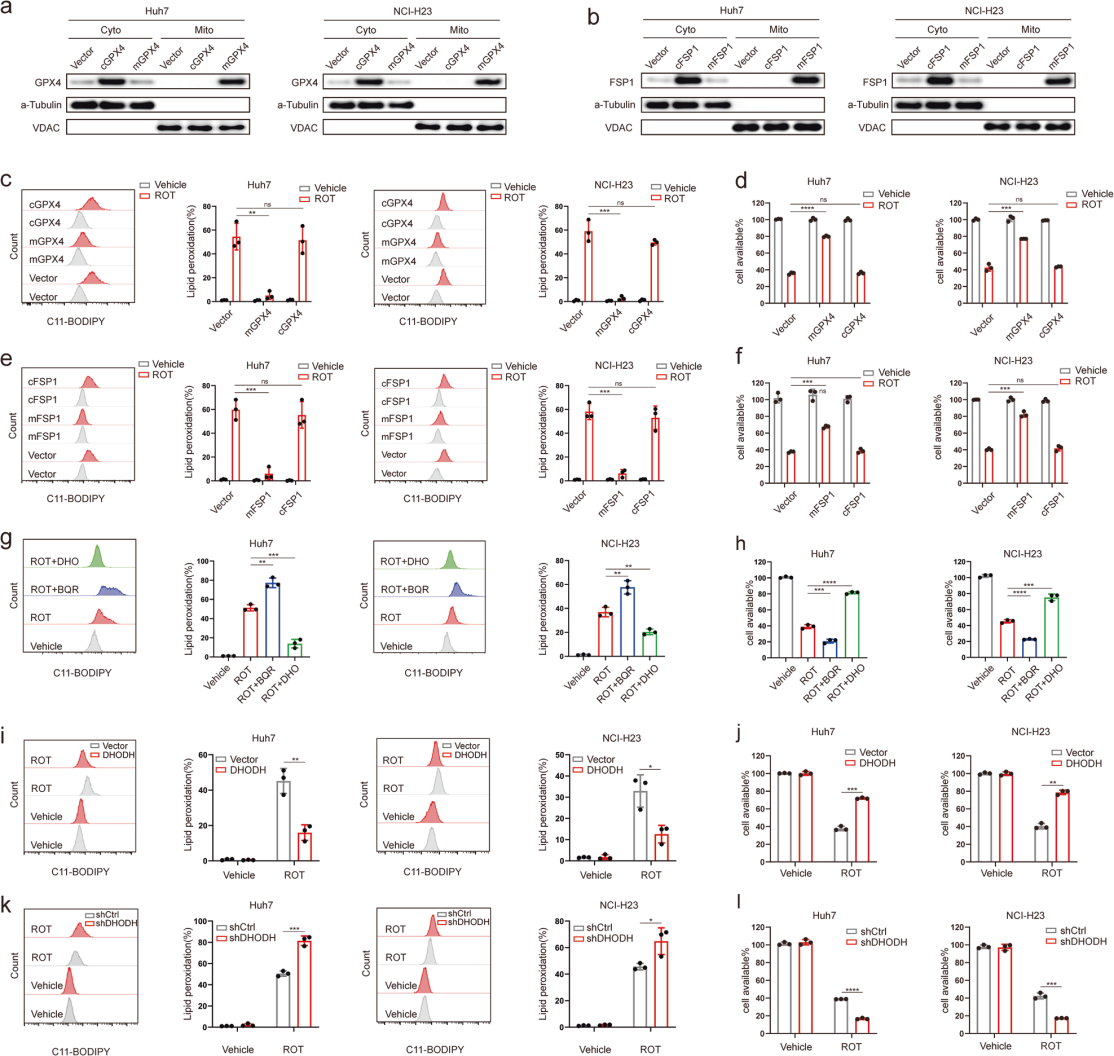
MCI acts in parallel with mitochondrial GPX4 and DHODH to suppress mitochondrial ferroptosis. **a** GPX4 protein levels in cytosolic and mitochondrial fractions from mitoGPX4 or cytoGPX4 overexpression Huh7 or NCI-H23 cells. **b** FSP1 protein levels in cytosolic and mitochondrial fractions from mitoFSP1 or cytoFSP1 overexpression Huh7 or NCI-H23 cells. **c** Lipid peroxidation levels in mitoGPX4(mGPX4) or cytoGPX4(cGPX4) overexpressing Huh7 or NCI-H23 cells treated with ROT (50 μM). **d** Cell viability in mGPX4 or cGPX4 overexpression Huh7 or NCI-H23 cells treated with ROT (50 μM). **e** Lipid peroxidation levels in mitoFSP1(mFSP1) or cytoFSP1 (cFSP1)-overexpressing Huh7 or NCI-H23 cells treated with ROT (50 μM). **f** Cell viability of mFSP1 or cFSP1 overexpression Huh7 or NCI-H23 cells treated with ROT (50 μM). **g** Lipid peroxidation levels in Huh7 or NCI-H23 cells treated with ROT (50 μM) with or without BQR (4 μM) or DHO (100 μM). **h** Cell viability in Huh7 or NCI-H23 cells treated with ROT with or without BQR(4 μM) or DHO (100 μM). **i** Lipid peroxidation levels in DHODH overexpression Huh7 or NCI-H23 cells treated with ROT (50 μM). **j** Cell viability of DHODH overexpression Huh7 or NCI-H23 cells treated with ROT(50 μM). **k** Lipid peroxidation levels in DHODH knockdown Huh7 or NCI-H23 cells treated with ROT (50 μM). **l** Cell viability in DHODH knockdown Huh7 or NCI-H23 cells treated with ROT(50 μM).

Cells have developed two distinct defense mechanisms to counteract ferroptosis. GPX4 employs reduced GSH to detoxify lipid peroxidation and inhibit ferroptosis. This includes both cytosolic-localized GPX4 (cytoGPX4) and mitochondria-localized GPX4 (mitoGPX4) [10, 11]. Additionally, FSP1 functions as an oxidoreductase that mainly operates on the plasma membrane, catalyzing the reduction of CoQ to CoQH2 [12, 13]. Is there a correlation between MCI, GPX4, and FSP1 in inhibiting ferroptosis based on two distinct classic defense mechanisms? To this end, we individually overexpressed mitoGPX4, cytoGPX4, mitoFSP1 (achieved through the introduction of a mitochondrial targeting sequence at the N-terminus of FSP1), and cytoFSP1 in multiple tumor cell lines (Fig. 5a, b). Our findings indicated that the overexpression of mitoGPX4 and mitoFSP1 significantly attenuated rotenone-induced ferroptosis in Huh7 and NCI-H23 cells. In contrast, cytoGPX4 and cytoFSP1 failed to reverse these effects (Fig. 5c–f and Supplementary Fig. S5a,b). It is noteworthy that FSP1 is not typically expressed in mitochondria. However, our results showed that mitoFSP1 is capable to inhibit ferroptosis within the mitochondria. Moreover, the protective effect of mitoGPX4 and mitoFSP1 overexpression extended to the inhibition of rotenone-induced ferroptosis across a variety of GPX4 low-expression cancer cell lines (Supplementary Fig. S5c-f).

Recent discoveries have highlighted the role of DHODH in inhibiting mitochondrial ferroptosis by channeling electrons from dihydroorotate acid (DHO) to CoQ, mediating the formation of CoQH2 [14]. Supplementation of cells with the DHODH substrate dihydroorotate (DHO) effectively protected cells against rotenone-induced ferroptosis (Fig. 5g, h and Supplementary Fig. S6a). Inactivation of DHODH increase ferroptosis sensitivity in cancer cells [14]. Treatment with DHODH inhibitor brequinar (BQR) exerted a more pronounced sensitizing effect on rotenone-induced ferroptosis in Huh7 and NCI-H23 cells (Fig. 5g, h and Supplementary Fig. S6a). Recent study suggest that pyrimidine synthesis also plays a role in ferroptosis regulation [48]. To distinguish between the roles of DHODH in pyrimidine synthesis versus CoQ reduction, uridine(50uM) was supplemented in all experiments using DHODH inhibitors. Overexpression of DHODH (Supplementary Fig. 6b) in Huh7 and NCI-H23 cells protected the cells against rotenone-induced ferroptosis (Fig. 5i, j and Supplementary Fig. S6d), whereas knockdown of DHODH (Supplementary Fig. S6c) had the opposite effect (Fig. 5k, l and Supplementary Fig. S6e), suggesting that the DHODH pathway protects cells against mitochondrial ferroptosis mediated by MCI inhibition by producing CoQH2. These results indicated that MCI acts in parallel with mitoGPX4 and DHODH to suppress mitochondrial ferroptosis.

### MCI inhibitors induce ferroptosis in vivo

**Fig. 6:**
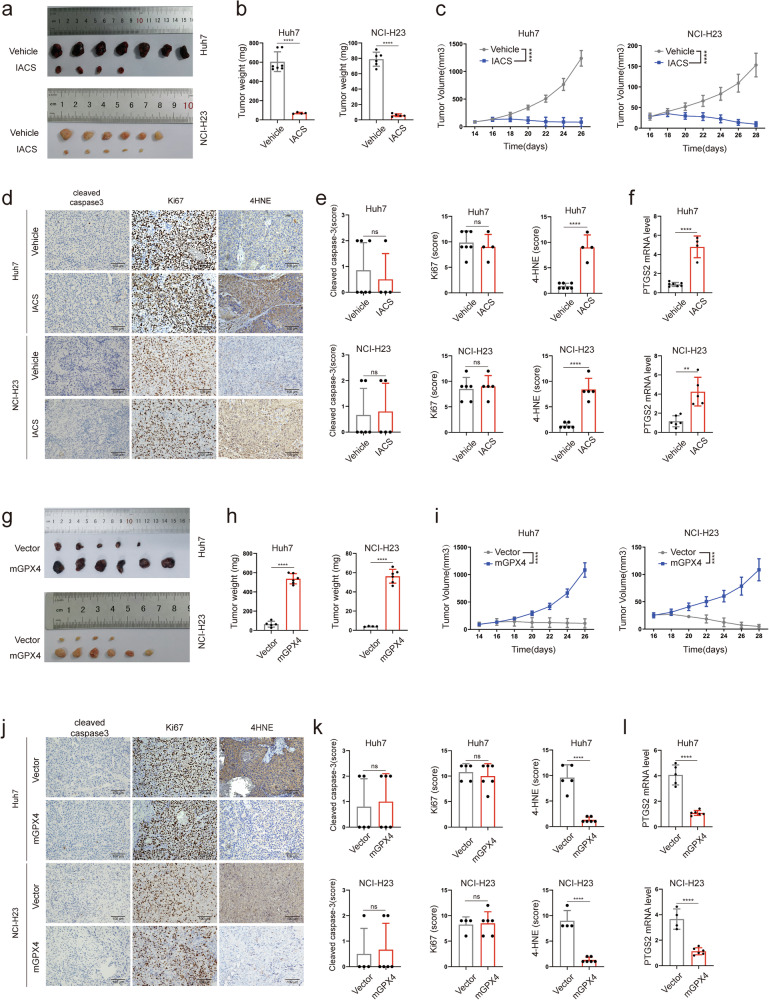
MCI inhibitors induce ferroptosis in vivo. **a** Tumor progression of Huh7 or NCI-H23 xenografts with IACS treatments over time. **b** Tumor weights of Huh7 or NCI-H23 xenograft tumors treated with IACS. **c** Tumor volumes of Huh7 or NCI-H23 xenografts treated with IACS over time. **d** Representative immunohistochemical images from Huh7 or NCI-H23 xenografts with the indicated treatments over time (200× magnification). **e** Immunohistochemistry scoring of cleaved caspase-3, ki67 and 4-HNE staining in Huh7 or NCI-H23 xenograft tumors with the indicated treatments. **f** PTGS2 mRNA levels in Huh7 and NCI-H23 xenograft tumors with indicated treatments. **g** Tumor progression of mitoGPX4 overexpression Huh7 or NCI-H23 xenografts treated with IACS over time. **h** Tumor weights of mitoGPX4-overexpression Huh7 or NCI-H23 xenograft tumors treated with IACS. **i** Tumor volumes of mitoGPX4 overexpression Huh7 or NCI-H23 xenografts treated with IACS over time. **j** Representative immunohistochemical images from mitoGPX4 overexpression Huh7 or NCI-H23 xenografts with the indicated treatments over time (200× magnification). **k** Immunohistochemistry scoring of cleaved caspase-3, ki67 and 4-HNE staining in mitoGPX4 overexpression Huh7 or NCI-H23 xenograft tumors with the indicated treatments. **l** PTGS2 mRNA levels in mitoGPX4 overexpression Huh7 or NCI-H23 xenograft tumors with indicated treatments.

Can in vivo experiments replicate similar results of in vitro ferroptosis? Animal models of subcutaneous tumors were established by subcutaneously injecting Huh7 and NCI-H23 cells into immune-deficient mice. IACS-010759 is endowed with the ability to induce ferroptosis in vitro. In vivo, we revealed that the administration of IACS-010759 effectively impeded the growth of Huh7 and NCI-H23 tumors in mice, which was maintained through the administration of pyruvate to maintain aspartate levels (Fig. 6a–c). It is pertinent to note that this effect was achieved without any change in the mice’s normal body weight (Supplementary Fig. S6f). Immunohistochemical analysis demonstrated that IACS-010759 did not have any noticeable influence on the staining for cleaved caspase-3 or Ki67 in these tumors (Fig. 6d, e). However, a prominent augmentation in staining for 4-HNE (a marker of lipid peroxidation) and PTGS2 mRNA levels was observed in the IACS-010759 treatment group (Fig. 6d–f).

Furthermore, tumors featuring overexpression of mitoGPX4 exhibited notable resistance to the effects of IACS-010759 (Fig. 6g–i), a phenomenon that did not lead to obvious changes in body weight (Supplementary Fig. S6g). Remarkably, the administration of IACS-010759 did not exert any discernible impact on staining for cleaved caspase-3 or Ki67 within the vector or mitoGPX4 overexpression group tumors (Fig. 6j, k). Nonetheless, a conspicuous increase in staining for 4-HNE and PTGS2 mRNA levels was observed within the vector group tumors, but not in the mitoGPX4 overexpression group (Fig. 6j–l). A significant increase in GPX4 staining was observed in mitoGPX4 overexpression group (Supplementary Fig. S6h, i). In summary, our in vivo investigations consistently demonstrated that inhibition of MCI effectively induced ferroptosis.

## Discussion

**Fig. 7:**
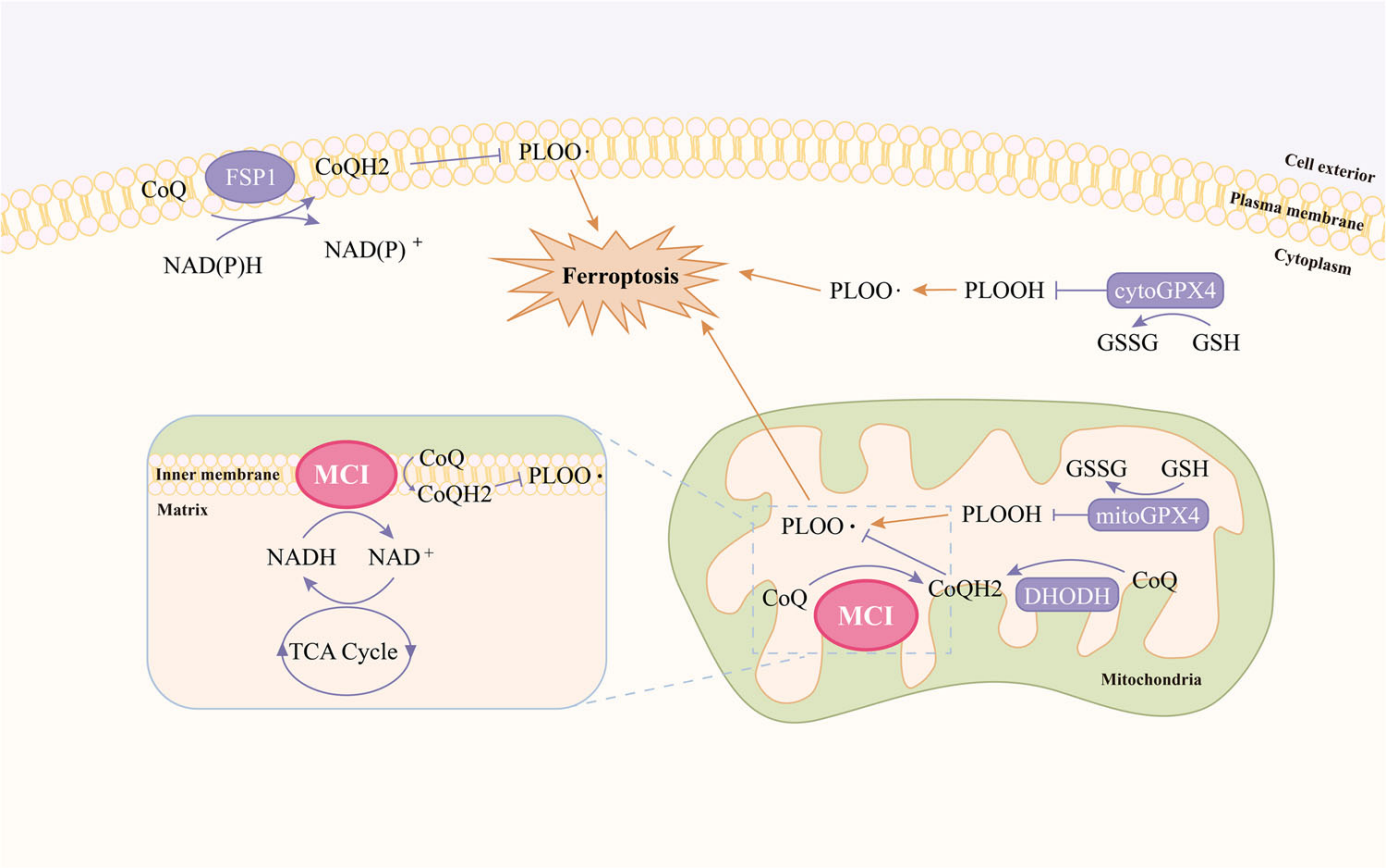
Model for MCI inhibits mitochondrial ferroptosis. See the main text for a detailed description. PLOOH phospholipid hydroperoxide, PLOO· phospholipid hydroperoxyl radical, GSSH oxidized glutathione, NAD(P)H reduced nicotinamide adenine dinucleotide (phosphate), NAD(P)+ oxidized nicotinamide adenine dinucleotide (phosphate), NADH reduced nicotinamide adenine dinucleotide, NAD+ oxidized nicotinamide adenine dinucleotide.

Ferroptosis, characterized by iron-dependent accumulation of phospholipid peroxide, is a regulated form of cell death. The susceptibility to ferroptosis is intricately linked to various biological processes, including redox homeostasis, iron metabolism, mitochondrial functionality, amino acid and lipid metabolism, as well as the biosynthesis of glutathione, phospholipids, NAD(P)H, and CoQH2 [2, 4]. Although recognized as playing a pivotal role in organ injuries, degenerative disorders, and as a potential target for cancer treatment [5, 7, 49], the complete mechanistic underpinnings of ferroptosis remain elusive. Cells have evolved a multifaceted array of defense mechanisms against ferroptosis. GPX4, present in both cytosol and mitochondria, constitutes a significant line of defense [10, 11]. Meanwhile, cytoGPX4 and FSP1 inhibit ferroptosis at the plasma membrane [12, 13]. In parallel, mitoGPX4 and DHODH inhibit ferroptosis within mitochondria [14]. In the present study, we systematically delineated an additional mechanism whereby MCI acts synergistically with mitoGPX4 and DHODH to inhibit mitochondrial ferroptosis by reducing CoQ to CoQH2 (Fig. 7).

Although FSP1 utilizes NADH to reduce CoQ to CoQH2 as an anti-ferroptotic measure [12], it remains unclear whether NADH can influence ferroptosis through mechanisms distinct from those of FSP1. Our data revealed that NADH not only inhibits ferroptosis induced by a ferroptosis inducer in wild-type cancer cells, but also exerts its anti-ferroptotic effects in FSP1 knockout cancer cells. This suggests an alternative NADH-mediated ferroptosis defense pathway independent of FSP1. Given NADH’s participation in various reactions, it is worth noting that the electron transport process in MCI consumes the highest levels of NADH. This led us to hypothesize that NADH-mediated ferroptosis defense might indeed be intertwined with MCI. Our findings indicate that administration of MCI inhibitors induces ferroptosis across a spectrum of GPX4 low-expression cancer cell lines, a phenomenon effectively reversed by ferroptosis inhibitors. MCI inhibitors cannot induce ferroptosis in tumor cell lines with high GPX4 expression, but they can increase the sensitivity of GPX4 high-expression cells to ferroptosis induced by a GPX4 inhibitor. Although MCI inhibition of cell proliferation by lowering intracellular aspartate levels has been reported [50], our data showed that supplementation with pyruvate or aspartate did not reverse the inhibition of MCI-induced ferroptosis. These findings may be related to GPX4 expression in different tumor cells.

NDI1, capable of replacing MCI in oxidizing NADH to NAD+ by donating electrons to CoQ without generating proton-motive force [46, 47], was employed in our study to highlight its potential to protect cancer cells from mitochondrial ferroptosis. We found that overexpression of NDI1 reverses MCI inhibitor-mediated ferroptosis, confirming that MCI inhibitor-mediated ferroptosis is not from unspecific effects of these inhibitors but is related to electron transportation in MCI. Furthermore, mitochondrial ferroptosis induced by GPX4 inhibitor or radiotherapy can be restored through the overexpression of NDI1 in cancer cells. These investigations underscores MCI’s role in protecting cancer cells against mitochondrial ferroptosis. MCI’s ability to reduce CoQ to CoQH2 through NADH donation [25] prompted us to explore whether MCI inhibitors triggered ferroptosis in tumor cells by diminishing CoQH2 levels. Our data conclusively revealed that MCI inhibitors drastically reduced the CoQH2 content within cancer cells. Notably, MCI inhibitor-induced ferroptosis was mitigated by the administration of mitochondria-specific CoQH2 analogs. These findings substantiate the role of MCI in inhibiting mitochondrial ferroptosis through the reduction of CoQ to CoQH2.

System XC transports extracellular cystine into the cell to facilitate glutathione synthesis [51].

MitoGPX4 employs GSH as a cofactor to catalyze the reduction of mitochondrial lipid peroxides to alcohols, thus protecting cells from mitochondrial ferroptosis [10, 11]. Similarly, DHODH plays a role in decomposing lipid peroxides within mitochondria by reducing CoQ to CoQH2 across the mitochondrial inner membrane, consequently inhibiting ferroptosis within the mitochondria [14]. We investigated whether MCI’s suppression of mitochondrial ferroptosis is intertwined with the activities of mitoGPX4 and DHODH. Our study suggests that MCI, mitoGPX4, and DHODH constitute three major defense mechanisms to detoxify lipid peroxides in the mitochondria.

IACS-010759, a clinical-grade small-molecule inhibitor of MCI that has undergone Phase I clinical trials for relapsed/refractory acute myeloid leukemia and solid tumors [30], has demonstrated its capacity to induce ferroptosis and inhibit tumor proliferation in vivo in GPX4 low-expression cancer, as confirmed in our study. In summary, our findings firmly established that MCI, alongside mitoGPX4 and DHODH, operates in tandem to inhibit mitochondrial ferroptosis.

Administration of an MCI inhibitor has been reported to attenuate ferroptosis mediated by cysteine depletion in mouse embryonic fibroblasts (MEFs) [18]. It has also been found that the MCI inhibitor BAY 87-2243 increases cell ROS levels, stimulates lipid peroxidation and glutathione consumption, and leads to the co-death of necrotic and iron cells in melanoma [19]. Recently, MCI was found to regulate ferroptosis in LKB1-deficient cancers [21]. It seems contradictory. However, our experiments confirmed that the MCI inhibitor could not induce ferroptosis in cells with high GPX4 expression but only in cells with low GPX4 expression. Moreover, cells with low GPX4 expression could resist the effect of MCI inhibitors on ferroptosis after overexpression of GPX4. This suggests that MCI inhibitors may act inconsistently in various cellular environments.

Our study also raises an intriguing question of the potential involvement of other mitochondrial enzymes that contribute to CoQH2 production, such as mitochondrial complex II, in the regulation of ferroptosis. Further investigation is required to elucidate the role of mitochondrial complex II in ferroptosis. Ferroptosis plays a pivotal role in a multitude of organ injuries and degenerative pathologies, encompassing neurotoxicity [52], acute renal failure [53], hepatic injuries [54], cardiac injuries [8], Alzheimer’s disease [55], Huntington’s chorea [56], and Parkinson’s disease [57]. Consequently, pharmacological modulation of MCI activity, either by induction or inhibition, has substantial potential for treating tumors, ischemic organ injuries, and other degenerative conditions marked by extensive lipid peroxidation. The application of IACS-010759 and the development of novel MCI inhibitors present promising avenues for cancer therapy. Future exploration of MCI agonists or MCI enzyme products may yield valuable strategies to address various organ injuries and degenerative diseases.

## Supplementary information

Supplementary information

Original Data

## Data Availability

The datasets used and/or analysed during the current study are available from the corresponding author on reasonable request.
