# Supplementary material for: Inhibition of mitochondrial complex I induces mitochondrial ferroptosis by regulating CoQH2 levels in cancer
Source: Cell Death Dis. 2025 Apr 5;16(1):254. doi: 10.1038/s41419-025-07510-6 (PMC11971431; doi:10.1038/s41419-025-07510-6)
Supplement: Supplementary file 1 — Supplementary information [file 41419_2025_7510_MOESM1_ESM.docx]

**Supplemental information**

**Supplementary Figure**


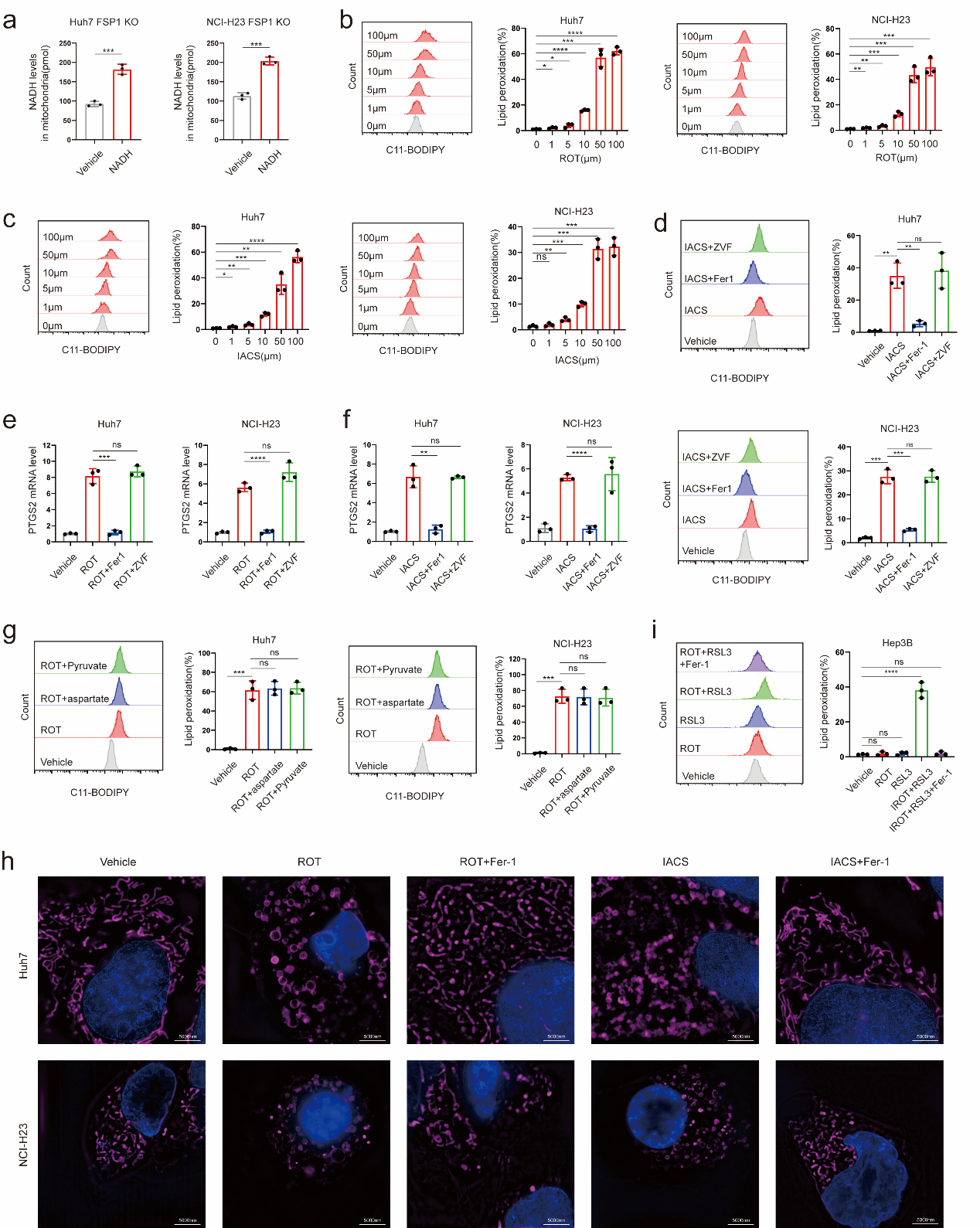


**Figure S1**

a, NADH levels in the mitochondria of the Huh7 or NCI-H23 FSP1-KO cells treated with NADH(1 mM) for 24 h.

b, c, Lipid peroxidation levels in Huh7 and NCI-H23 cells treated with ROT (a) or IACS-010759 (IACS) (b) at varying concentrations.

d, Lipid peroxidation levels in Huh7 or NCI-H23 cells treated with IACS (50 μM), following pretreatment with Fer-1(10 μM) or ZVF(10 μM).

e, f, PTGS2 mRNA levels in Huh7 or NCI-H23 cells treated with ROT (50 μM) (e) or IACS (50 μM) (f) after pretreatment with Fer-1 (10 μM) or ZVF (10 μM).

g, Lipid peroxidation levels in Huh7 or NCI-H23 cells treated with ROT (50 μM) with or without aspartate (100 μM) or pyruvate (0.5 mM).

h, SIMSTORM microscope analysis of mitochondrial morphology in Huh7 or NCI-H23 cells treated with ROT (50 μM) or IACS (50 μM) for 8 h after pretreatment with Fer-1 (10 μM).

i, Lipid peroxidation levels in Hep3B cells treated with ROT (50 μM) or RSL3(0.5 μM), following pretreatment with Fer-1(10 μM).


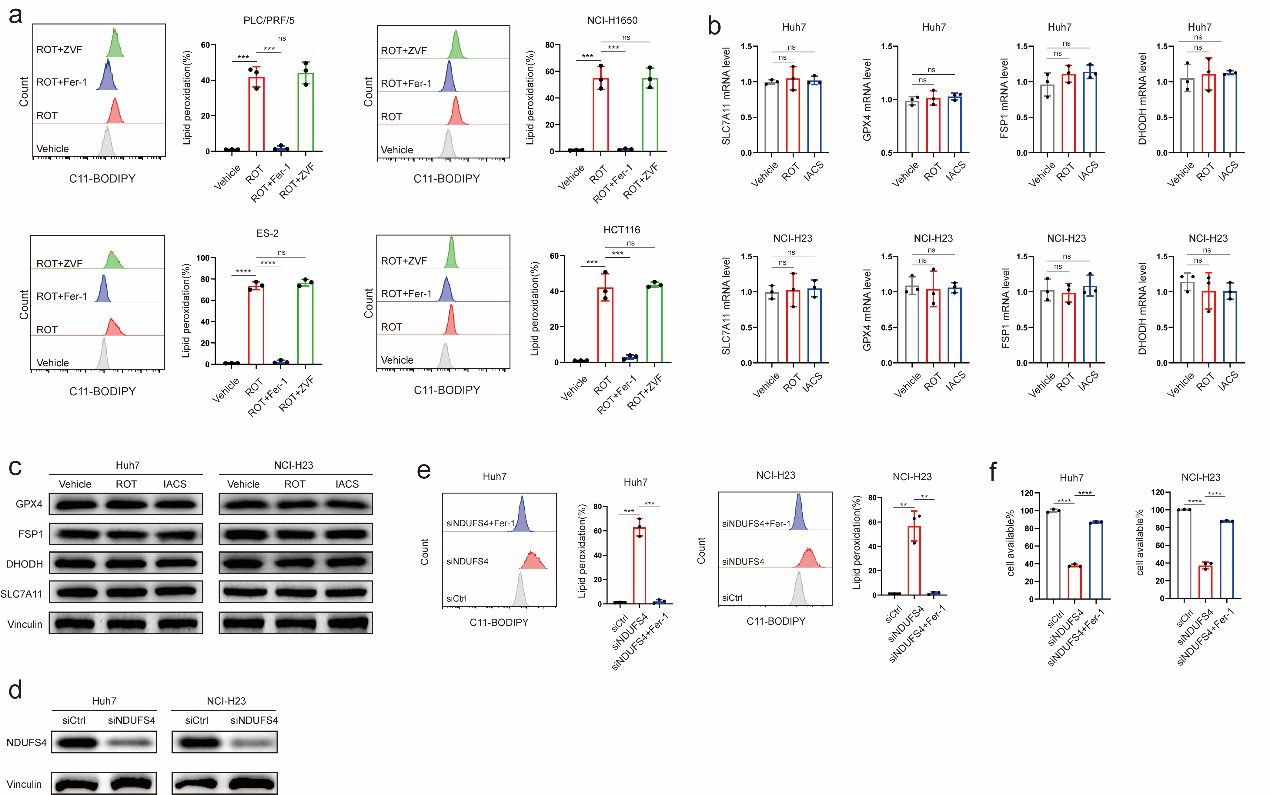


**Figure S2**

a, Lipid peroxidation levels in PLC/PRF/5, NCI-H1650, ES-2 and HCT-116 cells treated with ROT(50 μM) after pretreatment with Fer-1 (10 μM) or ZVF (10 μM).

b, SLC7A11, GPX4, FSP1, and DHODH mRNA levels in Huh7 and NCI-H23 cells treated with ROT (50 μM) or IACS (50 μM).

c, Protein levels of GPX4, FSP1, DHODH, and SLC7A11 in Huh7 and NCI-H23 cells treated with ROT (50 μM) or IACS (50 μM), as determined by western blotting.

d, NDUFS4 protein levels in NDUFS4 knockdown Huh7 or NCI-H23 cells.

e, Lipid peroxidation levels in NDUFS4 knockdown Huh7 or NCI-H23 cells treated with or without Fer-1 (10 μM).

f, Cell viability in NDUFS4 knockdown Huh7 or NCI-H23 cells treated with or without Fer-1 (10 μM).


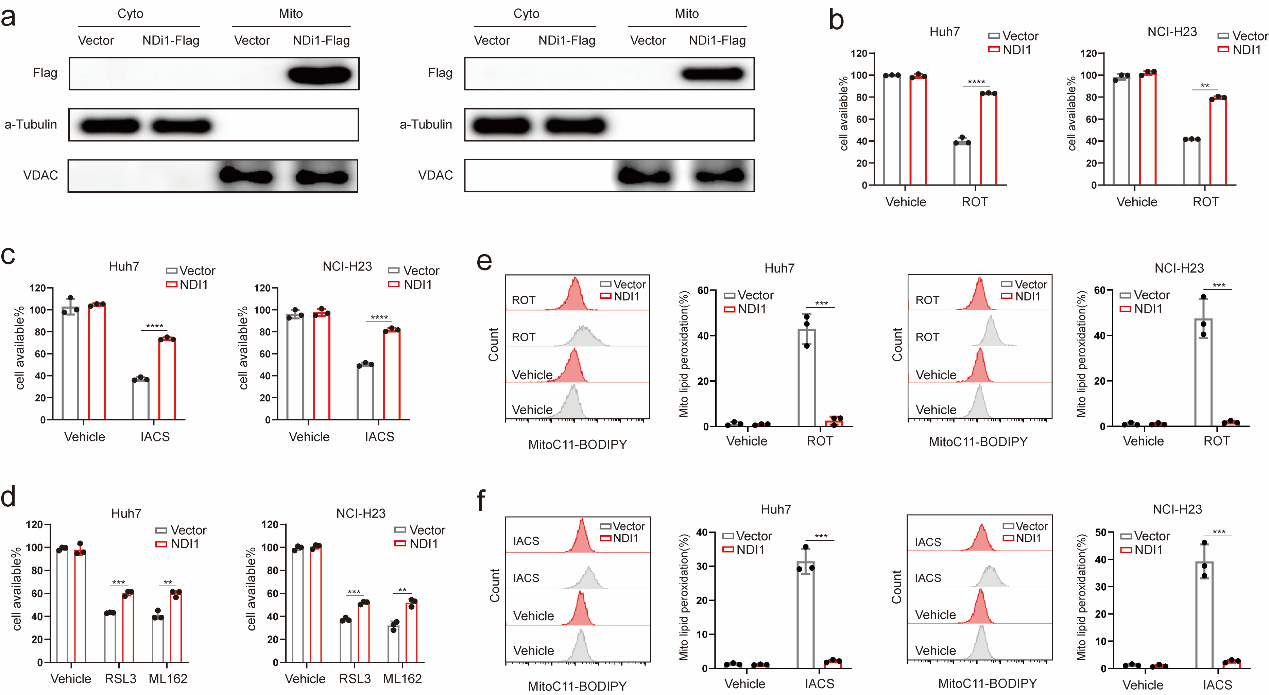


**Figure S3**

a, NDI1-Flag protein levels in cytosolic and mitochondrial fractions from Huh7 and NCI-H23 cells.

b, c, Cell viability of NDI1 overexpression Huh7 or NCI-H23 cells treated with ROT(50 μM) (b) or IACS(50 μM) (c).

d, Cell viability of NDI1 overexpression Huh7 or NCI-H23 cells treated with RSL3(1 μM) or ML162(1 μM) for 2 h.

e, f, Mitochondrial lipid peroxidation levels in NDI1 overexpression Huh7 or NCI-H23 cells treated with ROT (50 μM) (e) or IACS (50 μM) (f).


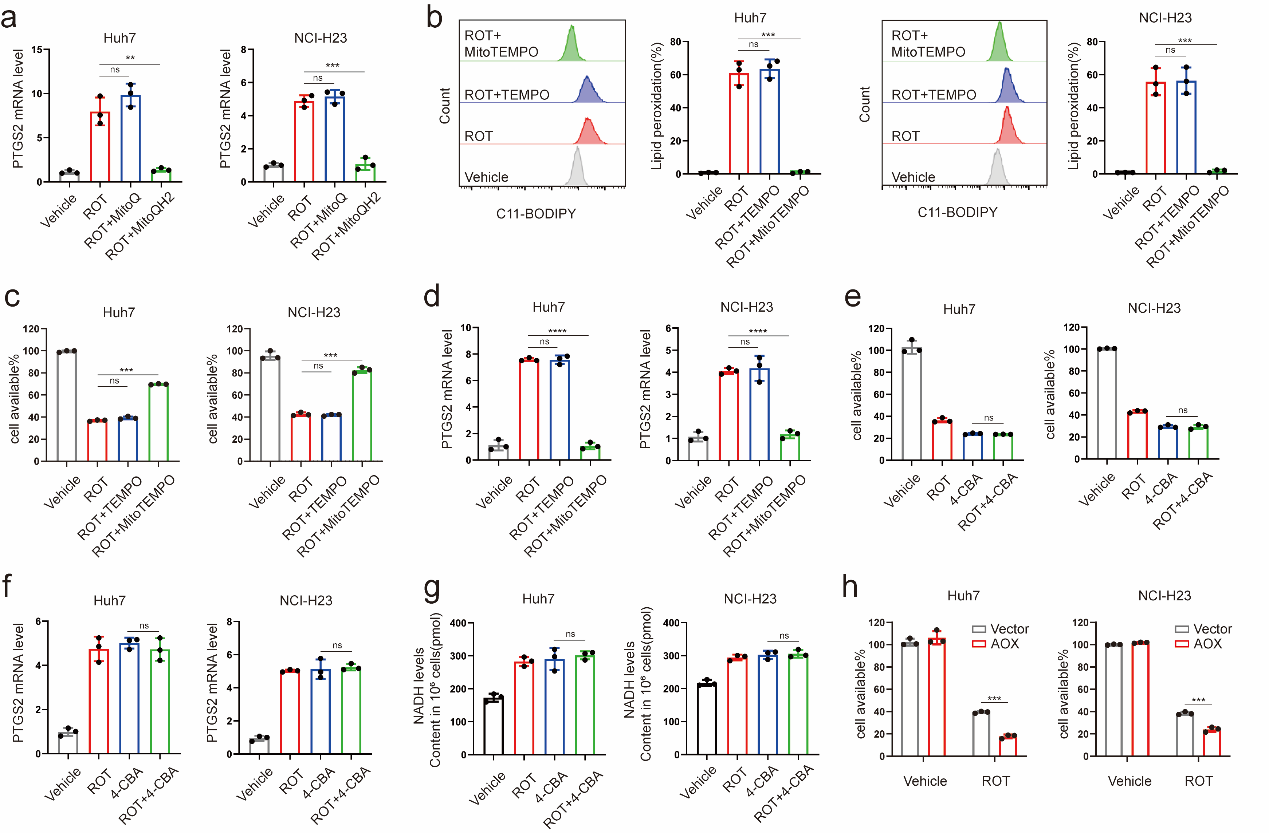


**Figure S4**

a, Cell viability in Huh7 or NCI-H23 cells treated with ROT, following pretreatment with MitoQ (5 μM) or MitoQH2 (5 μM).

b, Lipid peroxidation levels in Huh7 and NCI-H23 cells treated with ROT, following pretreatment with Tempo (5 μM) or MitoTempo (5 μM).

c, Cell viability of Huh7 or NCI-H23 cells treated with ROT, following pretreatment with Tempo (5 μM) or MitoTempo (5 μM).

d, PTGS2 mRNA levels in Huh7 and NCI-H23 cells treated with ROT, following pretreatment with Tempo (5 μM) or MitoTempo (5 μM).

e, Cell viability of Huh7 or NCI-H23 cells treated with ROT (50 μM) with or without 4-CBA (5 mM).

f, PTGS2 mRNA levels in Huh7 or NCI-H23 cells treated with ROT (50 μM) with or without 4-CBA (5 mM).

g, NADH levels in Huh7 or NCI-H23 cells treated with ROT (50 μM) with or without 4-CBA (5 mM).

h, Cell viability of AOX-overexpressing Huh7 or NCI-H23 cells treated with ROT (50 μM).


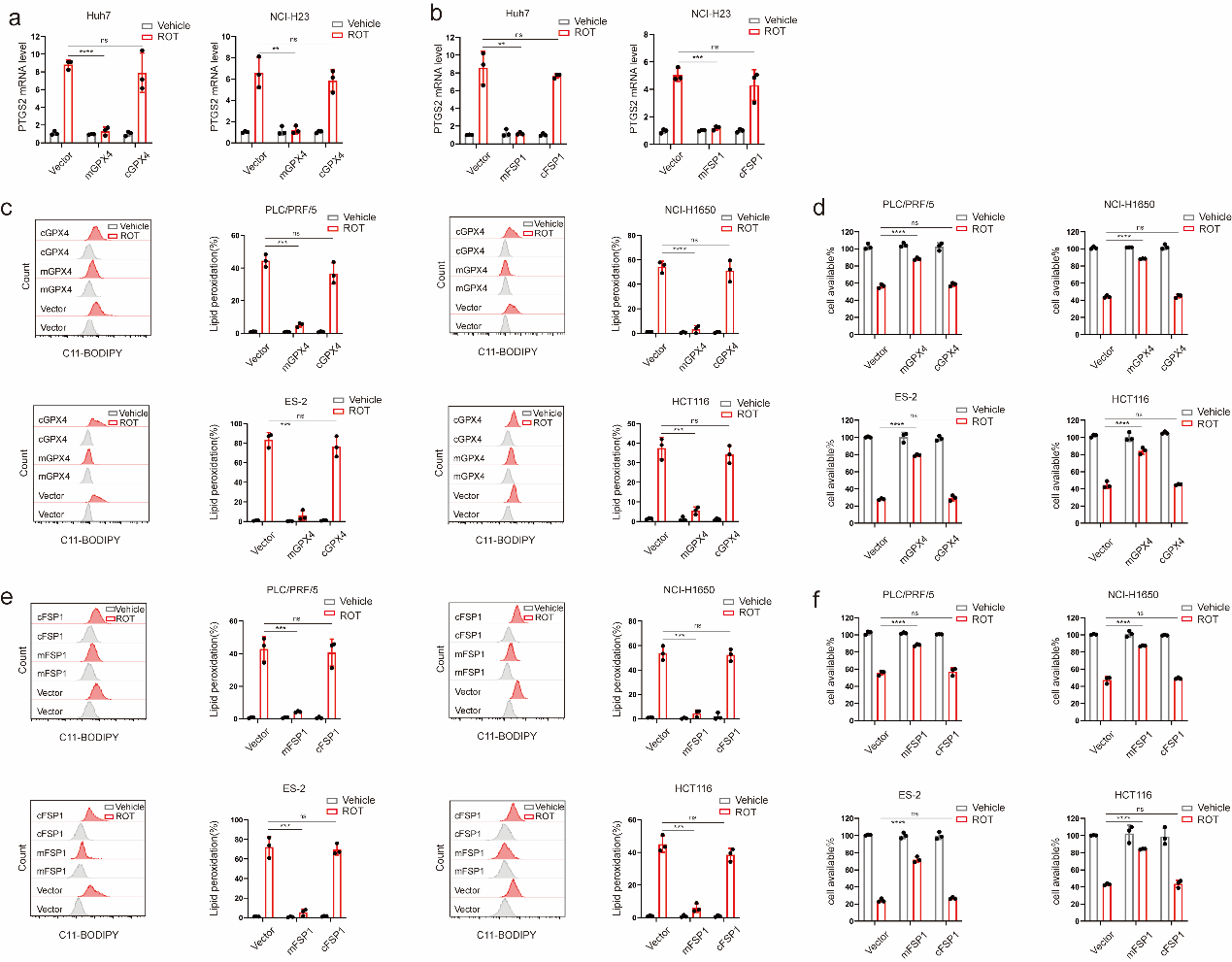


**Figure S5**

a, PTGS2 mRNA levels in mGPX4 or cGPX4 overexpression Huh7 or NCI-H23 cells treated with ROT (50 μM).

b, PTGS2 mRNA levels in mFSP1 or cFSP1 overexpression Huh7 or NCI-H23 cells treated with ROT (50 μM).

c, Lipid peroxidation levels in mGPX4 or cGPX4 overexpressing PLC/PRF/5, NCI-H1650, ES-2, and HCT-116 cells treated with ROT (50 μM).

d, Cell viability of mGPX4 or cGPX4 overexpression PLC/PRF/5, NCI-H1650, ES-2, and HCT-116 cells treated with ROT (50 μM).

e, Lipid peroxidation levels in mFSP1 or cFSP1 overexpressing PLC/PRF/5, NCI-H1650, ES-2, and HCT-116 cells treated with ROT (50 μM).

f, Cell viability of mFSP1 or cFSP1 overexpression PLC/PRF/5, NCI-H1650, ES-2, and HCT-116 cells treated with ROT (50 μM).


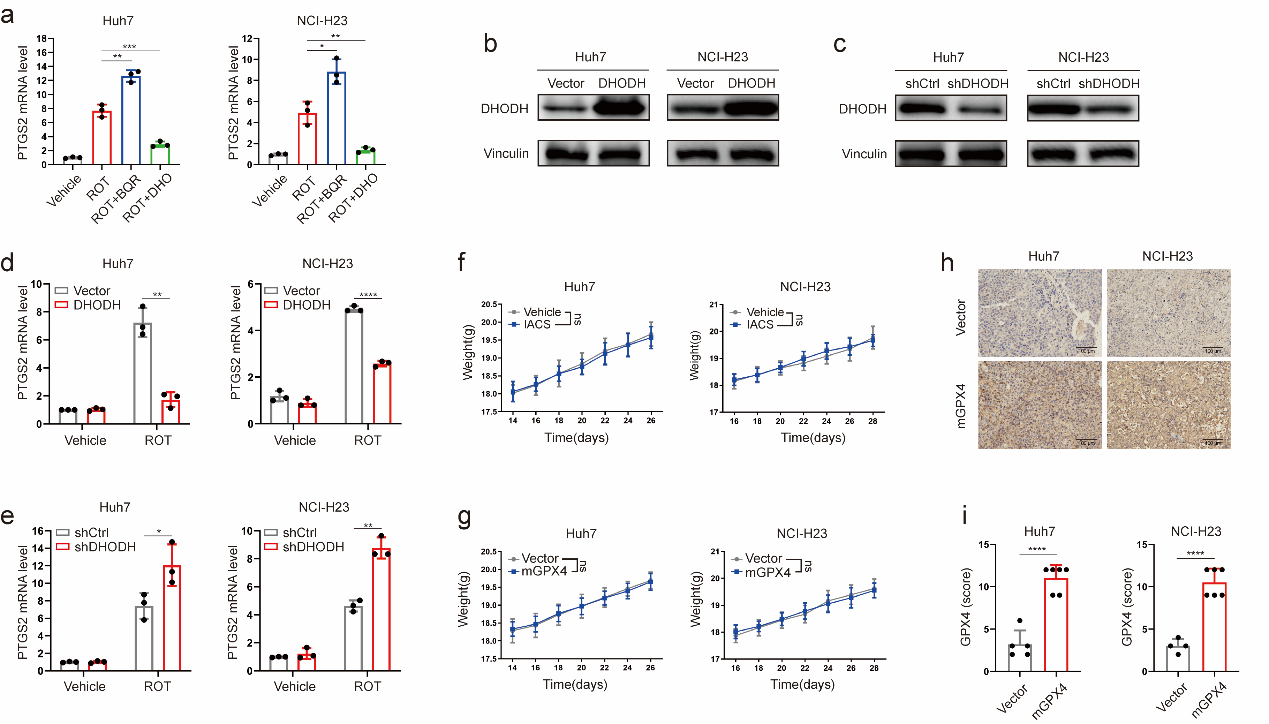


**Figure S6**

a, PTGS2 mRNA levels in Huh7 and NCI-H23 cells treated with ROT, with or without BQR (4 μM) or DHO (100 μM).

b, DHODH protein levels in DHODH overexpression Huh7 or NCI-H23 cells.

c, DHODH protein levels in DHODH knockdown Huh7 or NCI-H23 cells.

d, PTGS2 mRNA levels in DHODH-overexpressing Huh7 and NCI-H23 cells treated with ROT(50 μM).

e, PTGS2 mRNA levels in DHODH-knockdown Huh7 and NCI-H23 cells treated with ROT(50 μM).

f, Weight curve of mice with Huh7 or NCI-H23 xenograft tumors with the indicated treatments over time.

g, Weight curve of mice with mitoGPX4 overexpression Huh7 or NCI-H23 xenograft tumors with the indicated treatments over time.

h, Representative immunohistochemical images from mitoGPX4 overexpression Huh7 or NCI-H23 xenograft tumors (200× magnification).

i, Immunochemistry scoring of GPX4 staining in mitoGPX4 overexpression Huh7 or NCI-H23 xenograft tumors with indicated treatments.

**Supplementary Table 1：List of primers**

| **siRNA target sequences** | |
| --- | --- |
| NDUFS4 si | GAGGACTTCCACATGGAGATT |
| **shRNA target sequences** | |
| DHODH sh | CGATGGGCTGATTGTTACGAA |
| **Quantitative Reverse Transcription PCR primer sequences** | |
| PTGS2-F | CGGTGAAACTCTGGCTAGACAG |
| PTGS2-R | GCAAACCGTAGATGCTCAGGGA |
| SLC7A11-F | TCCTGCTTTGGCTCCATGAACG |
| SLC7A11-R | AGAGGAGTGTGCTTGCGGACAT |
| GPX4-F | ACAAGAACGGCTGCGTGGTGAA |
| GPX4-R | GCCACACACTTGTGGAGCTAGA |
| ACTB-F | CACCATTGGCAATGAGCGGTTC |
| ACTB-R | AGGTCTTTGCGGATGTCCACGT |
| **sgRNA oligos sequences** | |
| FSP1-sgRNA-F | CACCGGGAGATGGGGTCCCAGGTCT |
| FSP1-sgRNA-R | AAACAGACCTGGGACCCCATCTCCC |
