# Supplementary material for: Inhibition of mitochondrial complex I induces mitochondrial ferroptosis by regulating CoQH2 levels in cancer
Source: Cell Death Dis. 2025 Apr 5;16(1):254. doi: 10.1038/s41419-025-07510-6 (PMC11971431; doi:10.1038/s41419-025-07510-6)

Fig.1c

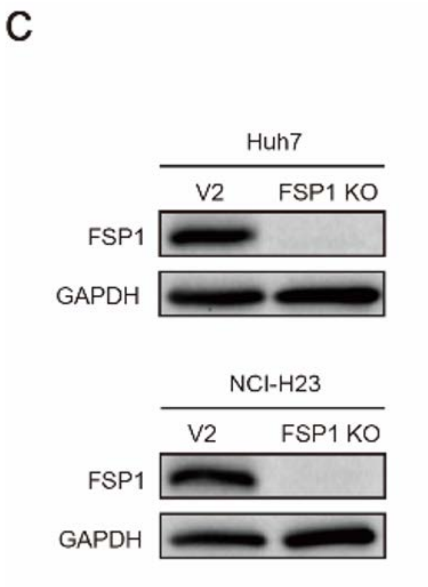
c
Huh7
V2
FSP1 KO
FSP1
GAPDH
NCI-H23
V2
FSP1 KO
FSP1
GAPDH

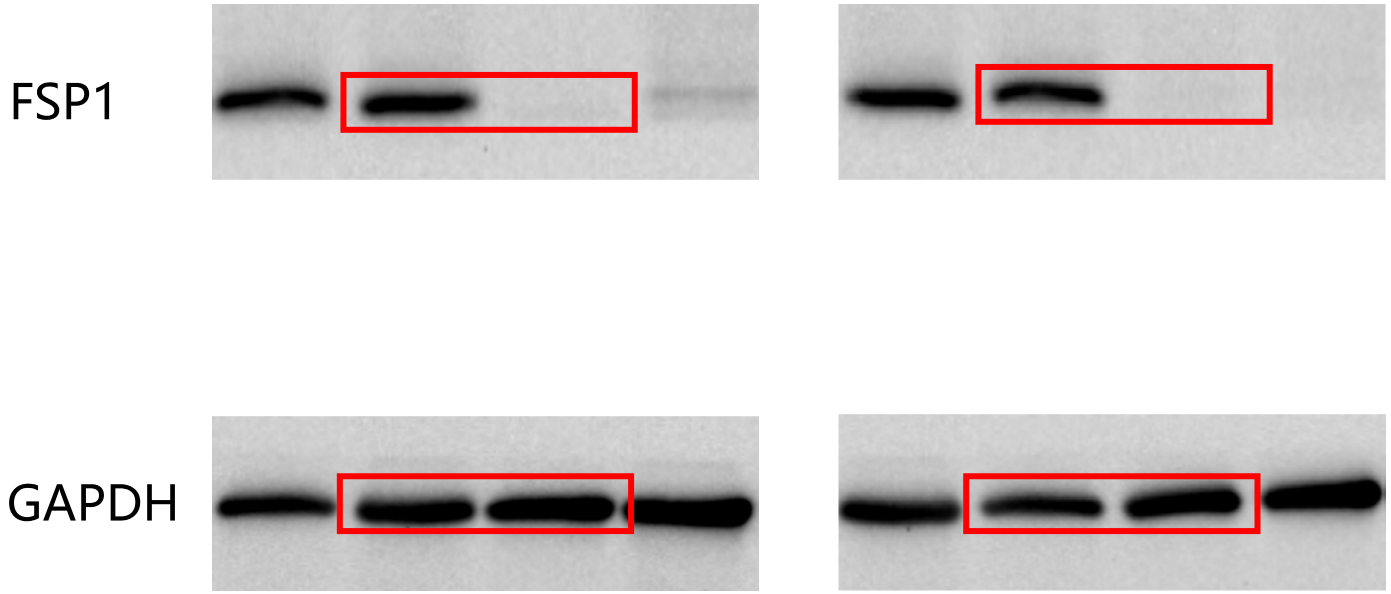
FSP1
GAPDH

Fig.S2d

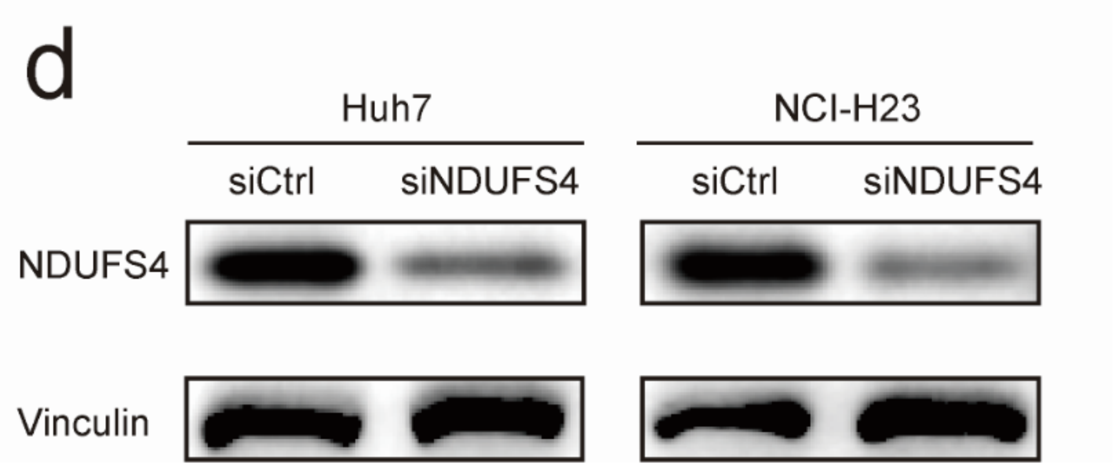
d
Huh7
NCI-H23
siCtrl
siNDUFS4
siCtrl
siNDUFS4
NDUFS4
Vinculin

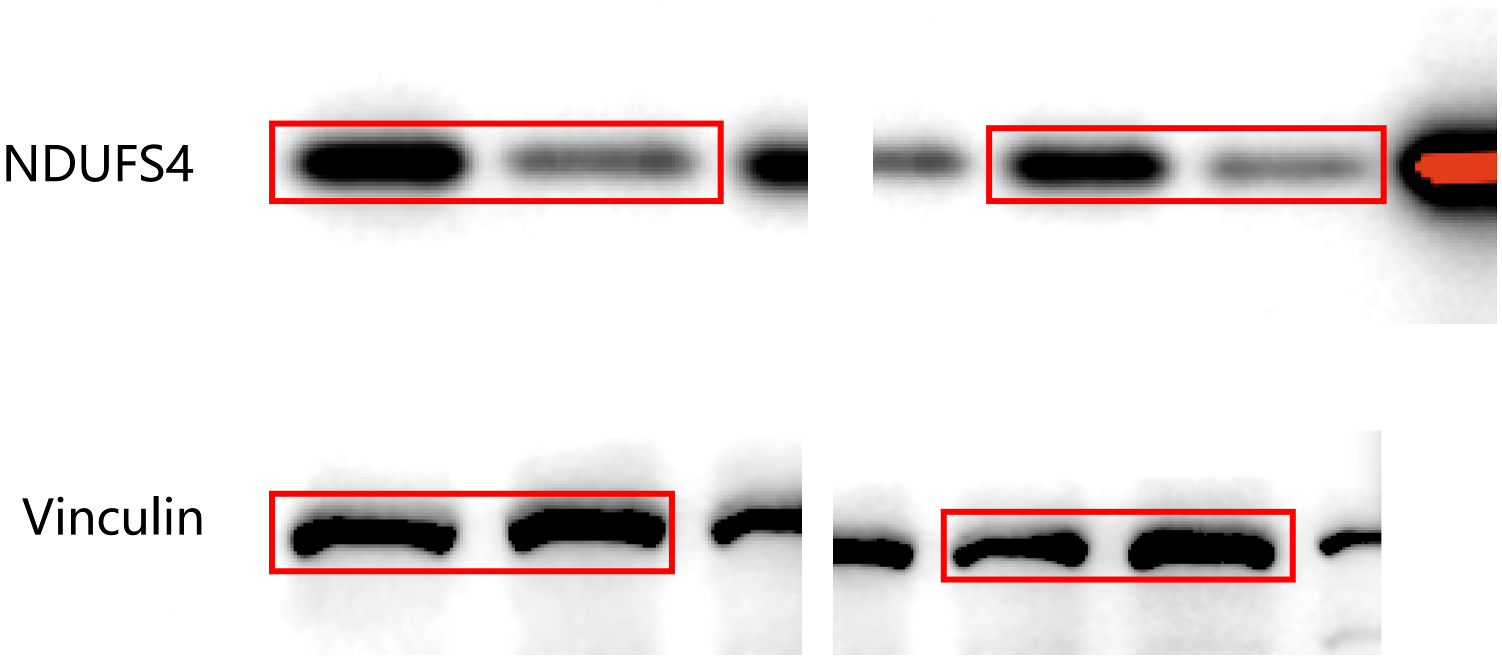
NDUFS4
Vinculin

Fig.2e

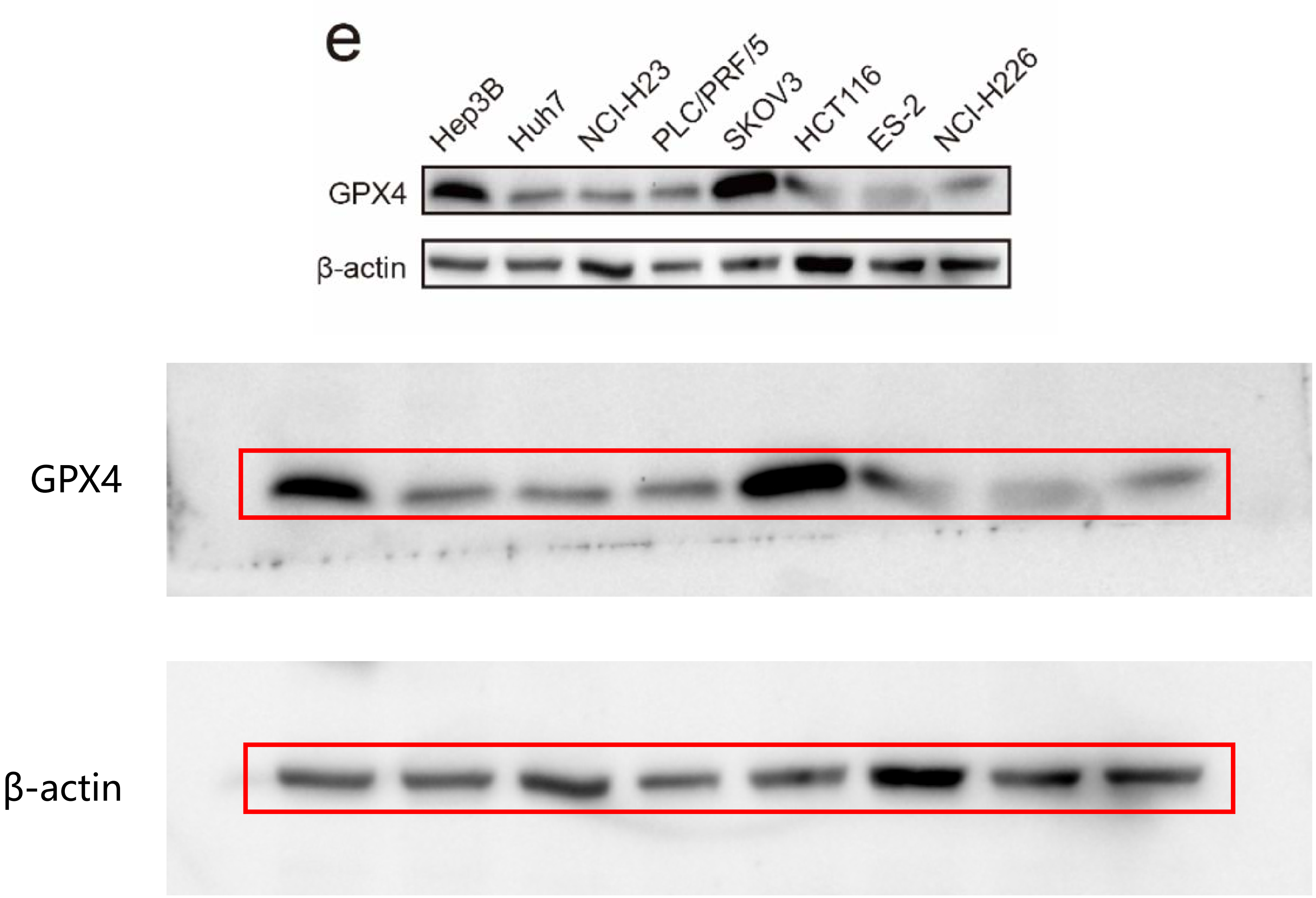
e
Hep3B
Huh7
NCI-H23
PLC/PRF/5
SKOV3
HCT116
ES-2
NCI-H226
GPX4
β-actin
GPX4
β-actin

Fig.5a,b

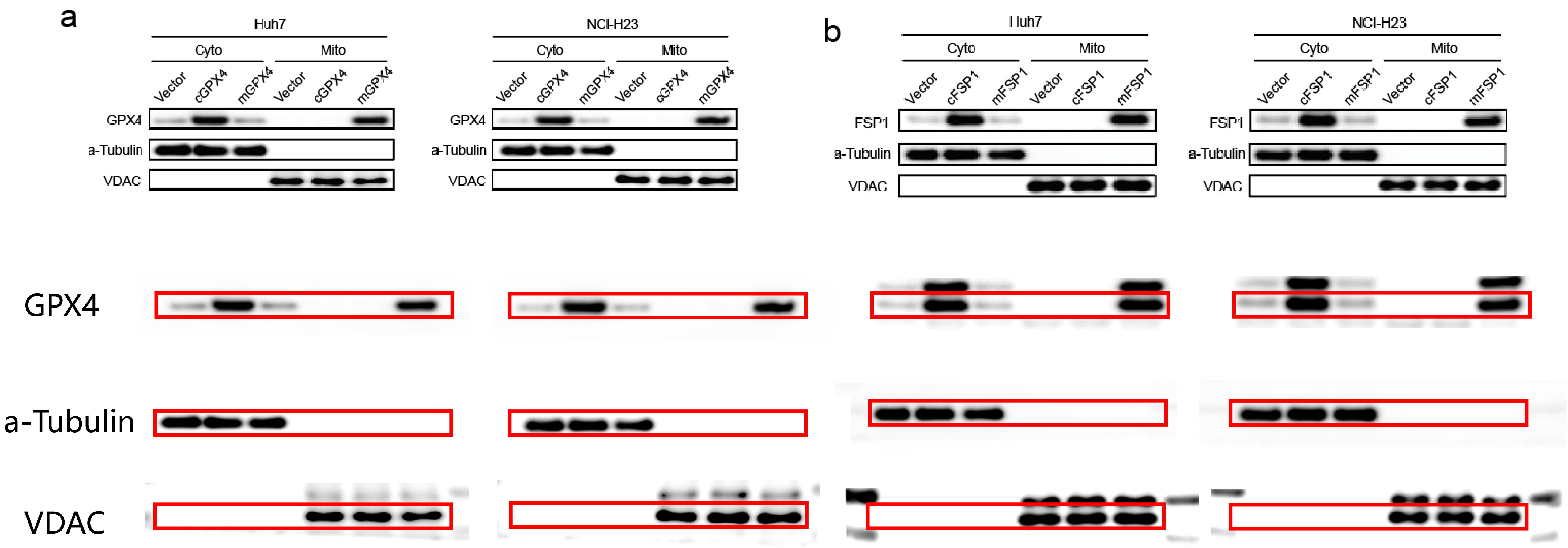
a
Huh7
Cyto
Mito
Vector
cGPX4
mGPX4
NCI-H23
GPX4
a-Tubulin
VDAC
b
Cyto
Mito
Vector
cFSP1
mFSP1
FSP1
GPX4
a-Tubulin
VDAC

Supplementary Fig.2c

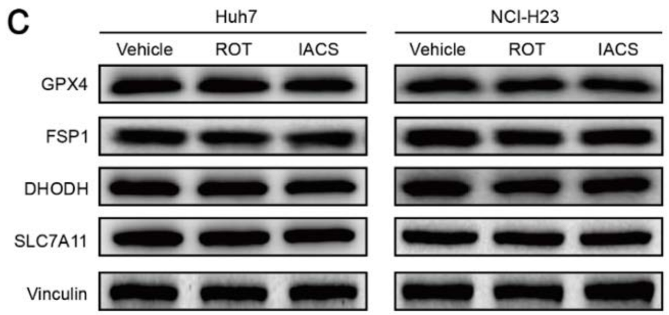

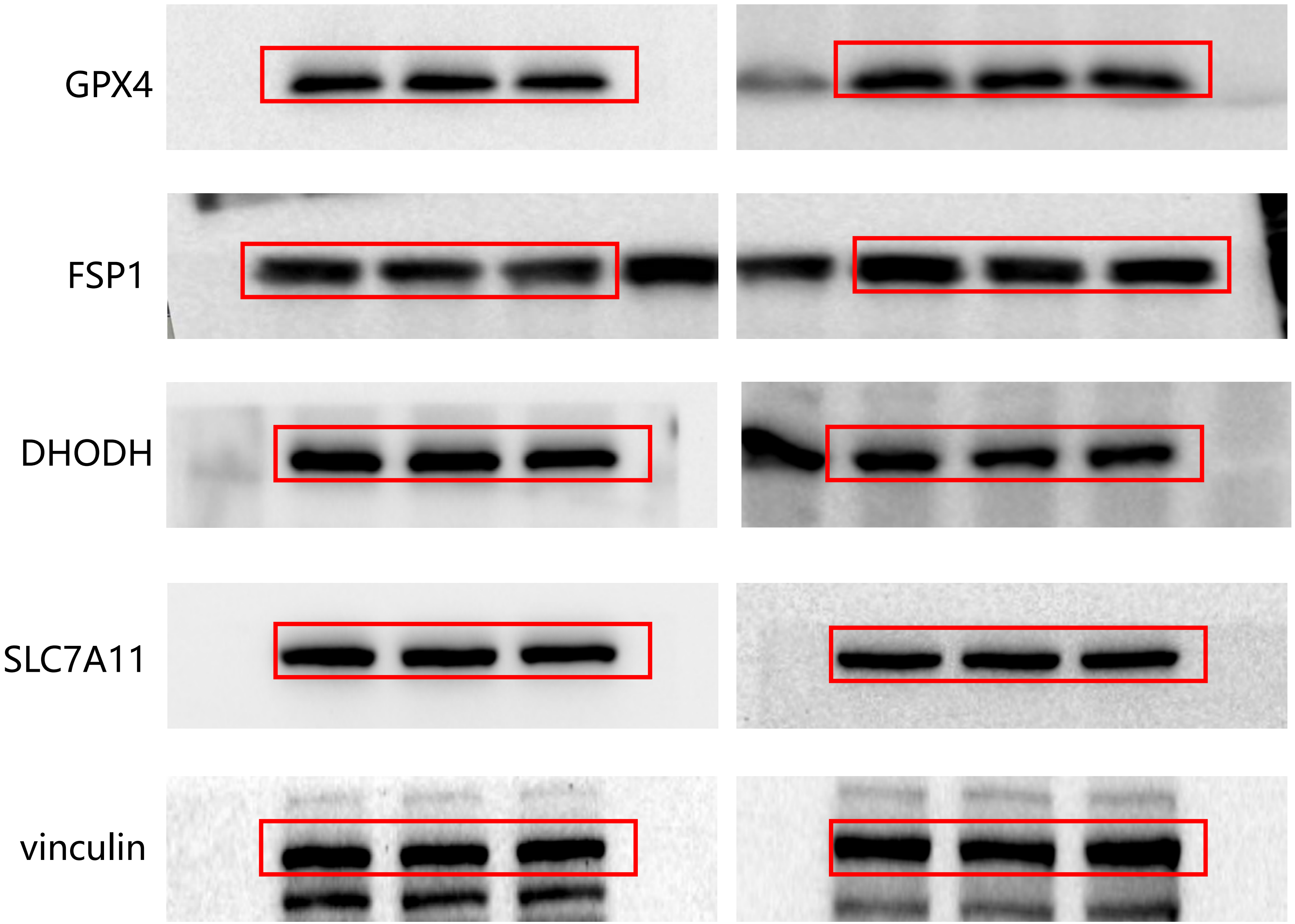

Supplementary Fig.3a

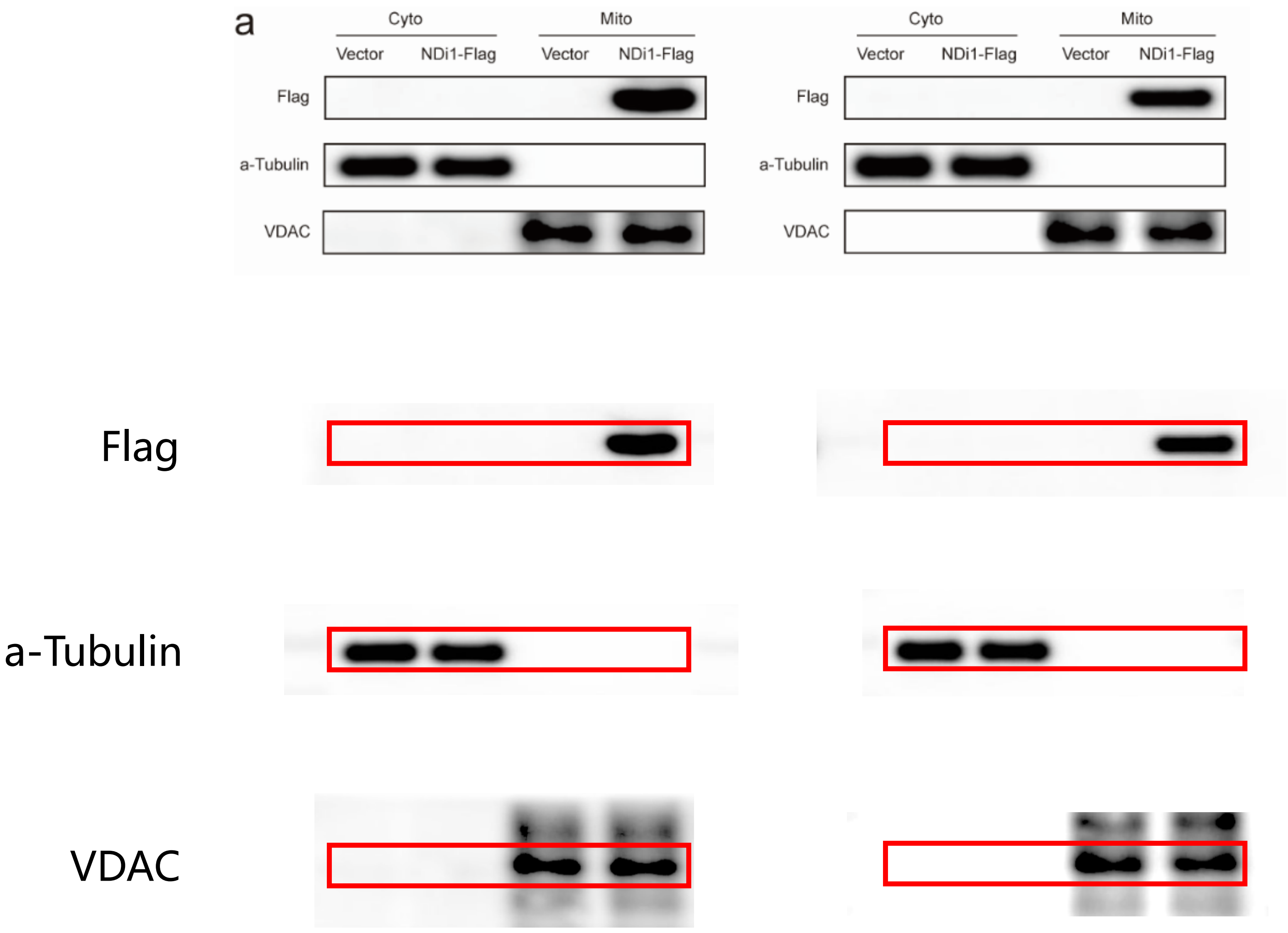

Supplementary Fig.6c,d

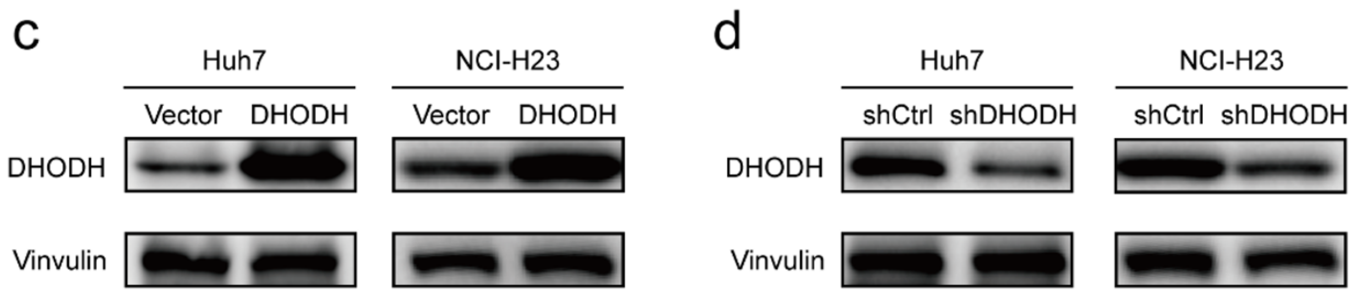

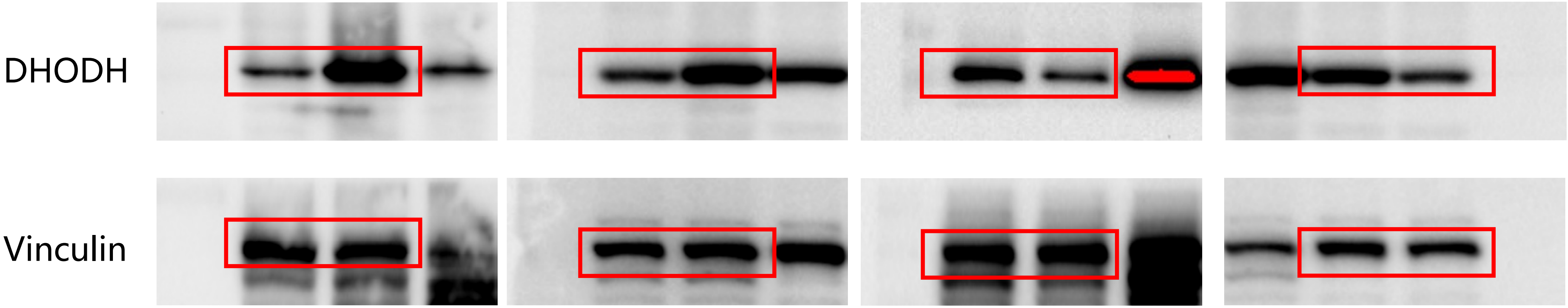

Supplement: Supplementary file 2 — Original Data [file 41419_2025_7510_MOESM2_ESM.pdf]
